# A human cell atlas of the pressure-induced hypertrophic heart

**DOI:** 10.1038/s44161-022-00019-7

**Published:** 2022-02-14

**Authors:** Luka Nicin, Sam Michael Schroeter, Simone Franziska Glaser, Ralf Schulze-Brüning, Minh-Duc Pham, Susanne S. Hille, Michail Yekelchyk, Badder Kattih, Wesley Tyler Abplanalp, Lukas Tombor, Oliver J. Müller, Thomas Braun, Benjamin Meder, Christoph Reich, Mani Arsalan, Tomas Holubec, Thomas Walther, Fabian Emrich, Jaya Krishnan, Andreas M. Zeiher, David John, Stefanie Dimmeler

**Affiliations:** 1 Institute for Cardiovascular Regeneration, Goethe University Frankfurt, Frankfurt, Germany; 2 German Center for Cardiovascular Research (DZHK), partner site Frankfurt Rhine-Main, Berlin, Germany; 3 Cardiopulmonary Institute, Goethe University Frankfurt, Frankfurt, Germany; 4 Cardiac Metabolism Group, Department of Cardiology, Goethe University Frankfurt, Frankfurt, Germany; 5 Department of Internal Medicine III, University Hospital Schleswig-Holstein, Kiel, Germany; 6 German Center for Cardiovascular Research (DZHK), partner site Hamburg/Kiel/Lübeck, Berlin, Germany; 7 Department of Cardiac Development and Remodelling, Max Planck Institute for Heart and Lung Research, Bad Nauheim, Germany; 8 Institute for Cardiomyopathies, University Hospital Heidelberg, Heidelberg, Germany; 9 German Center for Cardiovascular Research (DZHK), partner site Heidelberg/Mannheim, Berlin, Germany; 10 Department of Cardiovascular Surgery, Goethe University Hospital, Frankfurt, Germany

**Keywords:** Valvular disease, Translational research, Data integration

## Abstract

Pathological cardiac hypertrophy is a leading cause of heart failure, but knowledge of the full repertoire of cardiac cells and their gene expression profiles in the human hypertrophic heart is missing. Here, by using large-scale single-nucleus transcriptomics, we present the transcriptional response of human cardiomyocytes to pressure overload caused by aortic valve stenosis and describe major alterations in cardiac cellular crosstalk. Hypertrophied cardiomyocytes had reduced input from endothelial cells and fibroblasts. Genes encoding Eph receptor tyrosine kinases, particularly *EPHB1*, were significantly downregulated in cardiomyocytes of the hypertrophied heart. Consequently, EPHB1 activation by its ligand ephrin (EFN)B2, which is mainly expressed by endothelial cells, was reduced. EFNB2 inhibited cardiomyocyte hypertrophy in vitro, while silencing its expression in endothelial cells induced hypertrophy in co-cultured cardiomyocytes. Our human cell atlas of the hypertrophied heart highlights the importance of intercellular crosstalk in disease pathogenesis and provides a valuable resource.

## Abstract (web summary)

Using large-scale single-nucleus transcriptomics, Nicin et al. report insights into human cardiac hypertrophy, caused by pressure overload, at single-cell resolution. The authors show that intercellular communication, particularly via the Eph receptor tyrosine kinase EPHB1, is impaired in human cardiomyopathy.

## Main

The pathophysiology of cardiac hypertrophy is multifactorial and is accompanied by the dysregulation of various signaling pathways contributing to cardiac dysfunction and heart failure^1,2^. Initial studies focused on the hypertrophic response of cardiomyocytes to pressure overload, which has meanwhile been deeply characterized. In the last years, the interplay of cardiomyocytes with non-parenchymal cells in the heart, such as endothelial cells, fibroblasts and immune cells, gained increasing attention. Particularly, cardiomyocyte–endothelial cell crosstalk is important for cardiac development and for the coordinated response to injury^3^. For example, cardiac ischemia or pressure overload induces the expression of vascular endothelial growth factor (VEGF)A in cardiomyocytes to induce endothelial cell proliferation and angiogenic responses^4^. On the other hand, endothelial cells provide so-called ‘angiocrine’ factors, which are important for tissue repair and regeneration^5^. A deeper understanding of the multicellular composition of and molecular processes carried out by the full repertoire of vascular, cardiac and invading immune cells in human disease, however, is lacking.

Single-cell RNA sequencing or single-nucleus RNA sequencing (snRNA-seq) provide insights into the transcriptome of individual cells and are exquisitely useful to gain detailed knowledge of cellular signatures and disease-related alterations in humans. The technology has provided intriguing insights into the heterogeneity within cell populations during development^6^ and has disclosed cellular responses in experimental models of myocardial infarction and to pro-fibrotic and pro-hypertrophic stimuli (for example, refs. ^7–9^). Recent studies now provide insights into the transcriptional heterogeneity of the healthy human heart at single-cell resolution^10,11^. However, single-cell analyses of the diseased human heart are so far sparse and focused on end-stage heart failure^12,13^.

Here, we provide an analysis of the human hypertrophied heart of patients suffering from aortic valve stenosis (AS), which discloses insights into the transcriptional adaptation of cardiomyocytes and the impact on interactions with other cell types in the diseased hypertrophied heart.

## Results

### Data integration and cell annotation

**Fig. 1:**
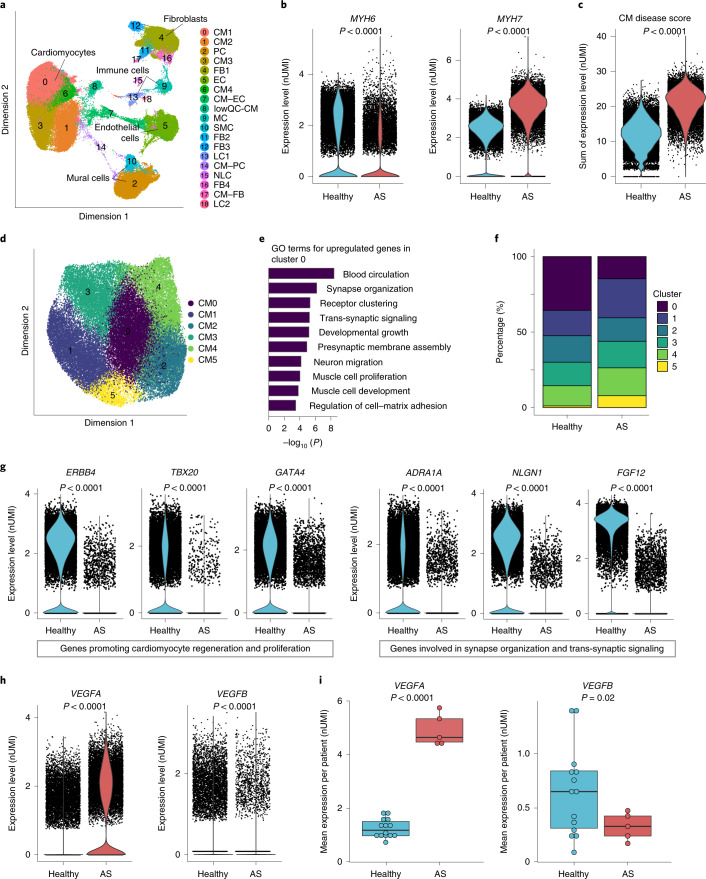
Dysregulated genes in cardiomyocytes from hypertrophied hearts. **a**, Representative uniform manifold approximation and projection (UMAP) plot after snRNA-seq and integration of data from cardiac tissues from the septum of 14 control hearts and five patients with AS and hypertrophy. A total of 88,536 nuclei were pooled. Nineteen cell clusters were identified: CM, EC, FB, PC, SMC, LC, MC and NLC. Three clusters had shared gene expression profiles (CM–EC, CM–PC and CM–FB), and one cluster was of low quality (lowQC-CM). **b**, Violin plots for the MYH isoforms *MYH6* and *MYH7 shown as number of unique molecular identifiers (nUMI)*. **c**, The cardiomyocyte disease score was calculated by the expression of ten established cardiomyocyte disease and stress markers per cell (*NPPA*, *NPPB*, *MYH7*, *MYH7B*, *XIRP2*, *CMYA5*, *ANKRD1*, *TNNI3*, *ACTA1* and *PFKP*). **d**, Unbiased reclustering of the four cardiomyocyte clusters, shown as a UMAP plot, revealed six subclusters of cardiomyocytes (CM0–CM5). **e**, GO term analysis for the genes upregulated in cluster 0 versus those in all other clusters. **f**, Quantitative analysis of the distribution of cardiomyocytes in the subclusters from **d** respective to their origin in percent. **g**, Violin plots for genes expressed in cardiomyocytes. **h**, Violin plots for *VEGFA* and *VEGFB* expression in cardiomyocytes. **i**, Box plots illustrate expression of *VEGFA* and *VEGFB* in cardiomyocytes shown for individual patients (*n* = 14 healthy individuals, *n* = 5 patients with AS; log_2_-transformed and normalized UMI counts, visualized as median and 25th and 75th percentiles, with whiskers indicating maximal and minimal values). Violin plots (**b**,**c**,**g**,**h**) represent log_2_-transformed and normalized UMI counts. Adjusted *P* values based on Bonferroni correction using all genes in the dataset to compare expression in violin plots were calculated with the Seurat function ‘FindAllMarkers’ using ‘bimod’ as the statistical test (**b**,**c**,**g**,**h**). Normal distribution was assessed using the Kolmogorov–Smirnov test (**i**). Statistical analysis to compare two groups was performed using unpaired, two-sided Student’s *t*-tests (**i**).

**Extended Data Fig. 1:**
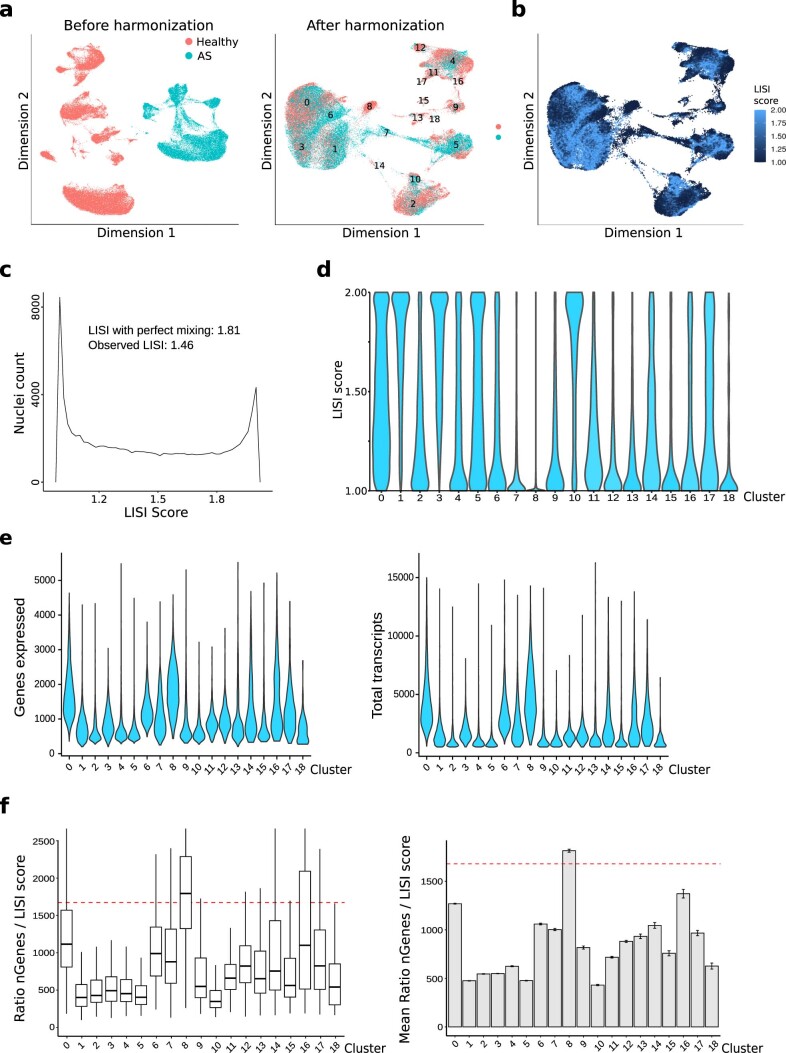
snRNA-seq dataset quality control (1). (**a**) Representative uniform manifold approximation and projection (UMAP) plots before (left panel) and after (right panel) harmonization of the two datasets (Healthy, n=14, vs. aortic valve stenosis=AS, n=5). (**b**) UMAP plot showing the local inverse Simpson’s index (LISI) integration score per nucleus, indicated by a color code (a score of 2 represents the ideal integration of the datasets). (**c**) Density plot for the distribution of the LISI integration scores per nucleus. (**d**) Violin plot for the LISI integration score per cell-cluster. (**e**) Violin plots of numbers of genes expressed per nucleus (top) and for the total amount of transcripts expressed per nucleus (bottom) in each cell-cluster. (**f**) Left: box plots for the ratio of the number of genes vs. the LISI score per cluster (visualized as median and 25th and 75th percentiles with whiskers indicating maximal and minimal values.). Right: bar graphs for the ratio of the number of genes vs. the LISI score per cluster given as mean ± s.e.m. The red lines indicate the threshold value calculated using the identify_outlier() function, where mean ratios above Q3+1.5 × IQR or below Q1-1.5 × IQR were considered as outliers and resulted in exclusion of the respective cluster (Q1 = first quartile, Q3 = third quartile, IQR = interquartile range). Solid lines indicate maxima and minima without outliers. Cells per cluster: 0=21313, 1=10960, 2=10818, 3=10644, 4=9752, 5=8284, 6=2811, 7=2283, 8=2108, 9=1937, 10=1707, 11=1328, 12=1289, 13=1036, 14=642, 15=518, 16=477, 17=455, 18=174.

**Extended Data Fig. 2:**
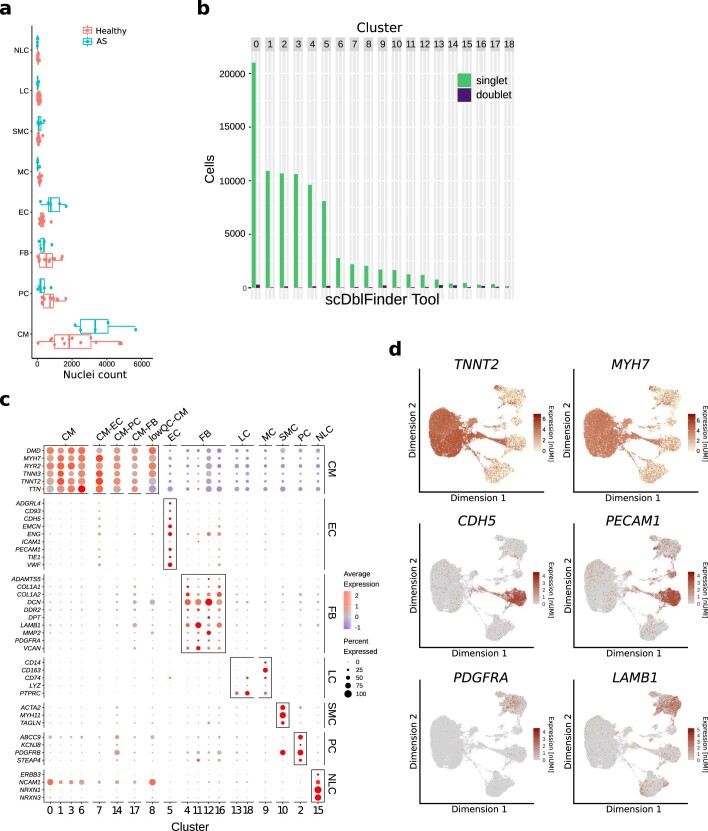
snRNA-seq dataset quality control (2). (**a**) Box plots for the distribution of annotated cell types according to condition; CM=cardiomyocytes; PC=pericytes; FB=fibroblasts; EC=endothelial cells; MC= monocytes; SMC=smooth muscle cells; LC=leukocytes; NLC=neuronal-like cells. Red= Healthy (n=14); blue= AS (n=5); visualized as median and 25th and 75th percentiles with whiskers indicating maximal and minimal values. (**b**) Bar plot for the number of singlets (green) and doublets (violet) per cluster identified by the bioinformatic tool scDblFinder. (**c**) Cell-type specific marker expression for all annotated clusters from panel (a). Markers shown are a combination of well-known cell-type markers and genes identified using the unbiased Seurat function ‘FindAllMarkers’. Colors of the dots reflect the fold change (natural log) of the respective gene and size reflects the percentage of nuclei expressing the marker. (**d**) FeaturePlots for the expression of TNNT2, MYH7 (cardiac markers), CDH5, PECAM1 (endothelial cell markers), PDGFRA and LAMB1 (fibroblast markers) in the human cardiac snRNA-seq dataset (log2 transformed and normalized UMI counts). Color represents the expression.

We analyzed organ location-matched cardiac tissue of patients with hypertrophy and healthy hearts. Briefly, samples were obtained from the cardiac septum of five patients with severe AS showing cardiac hypertrophy, and the resulting data were merged with a publicly available dataset of septal tissue from fourteen healthy hearts (Supplementary Tables 1 and 2). We used the algorithm ‘Harmony’, which allows accurate integration of single-cell sequencing data by projecting cells into a shared embedding by grouping cell types^14^. Unsupervised clustering with a total of 88,536 nuclei revealed 19 distinct clusters (Fig. 1a). Quality controls ensured that cells from healthy and hypertrophic hearts were well integrated and distributed in the clusters (Extended Data Fig. 1a–d). Analysis of the integration score, numbers of genes expressed per cell, total transcripts per cell, the ratio of the number of genes expressed versus the integration score and the cell type representation as well as identification of possible doublets ensures the high quality of both datasets and successful integration (Extended Data Figs. 1e,f and 2a,b). Using a combination of unbiased analysis and well-known cell type-specific gene markers, seven major cell types could be annotated, including cardiomyocytes (CM, four clusters), endothelial cells (EC, one cluster), fibroblasts (FB, four clusters), pericytes (PC, one cluster), smooth muscle cells (SMC, one cluster) and immune cells (leukocytes (LC) and monocytes (MC), three clusters) (Fig. 1a and Extended Data Fig. 2a). Expression of characteristic marker genes allowed us to annotate the cell populations (Extended Data Fig. 2c,d). For example, cardiomyocytes expressed genes encoding troponin (*TNNT2)*, myosin heavy chain (MYH) 7 (*MYH7*; Extended Data Fig. 2d), endothelial cells expressed genes encoding vascular endothelial (VE)-cadherin (*CDH5*) and CD31 (*PECAM1*; Extended Data Fig. 2d), and fibroblasts were characterized by *PDGFRA* (platelet-derived growth factor receptor α) and *LAMB1* (laminin subunit β1; Extended Data Fig. 2d) expression. One cluster contained neuronal markers (such as neurexins, *NRXN*) but did not express typical Purkinje cell-specific or other established neuronal cell-specific genes and was therefore annotated as containing ‘neuronal-like’ cells (NLC). Three other clusters expressed well-established cardiomyocyte markers but coexpressed either endothelial, fibroblast or pericyte markers. Because these clusters passed quality control and there was no evidence of doublets (Extended Data Fig. 2b), they were annotated as the ‘cardiomyocyte–endothelial-like cluster’ (CM–EC), the ‘cardiomyocyte–pericyte-like cluster’ (CM–PC) or the ‘cardiomyocyte–fibroblast-like cluster’ (CM–FB) (Fig. 1a and Extended Data Fig. 2c). Only one cluster (cluster 8; namely, low-quality cluster, ‘lowQC’) did not pass quality control (based on an outlier test; Methods) and, therefore, was excluded from further analysis to ensure well-characterized and high-quality cell cluster annotation for downstream analysis.

### Characterization of cardiomyocytes

**Extended Data Fig. 3:**
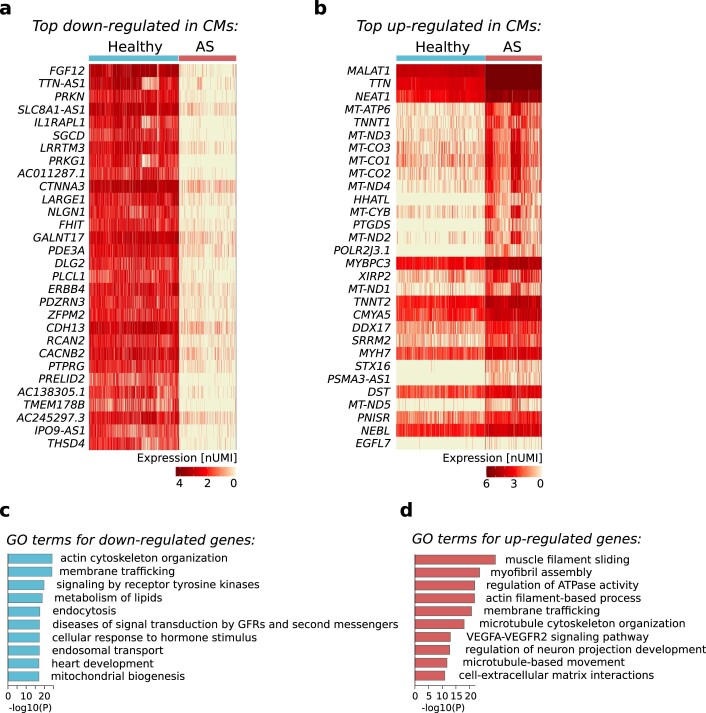
Disturbed gene expression signatures in hypertrophic cardiomyocytes. (**a**) Heatmap for the top 30 down-regulated genes in hypertrophied (AS) vs. healthy cardiomyocytes (log2 transformed and normalized UMI counts). Color reflects the expression. (**b**) Heatmap for the top 30 up-regulated genes in hypertrophied (AS) vs. healthy cardiomyocytes (log2 transformed and normalized UMI counts). Color represents the expression. (**c**) Gene ontology (GO) term analysis for the 2775 down-regulated genes in hypertrophied vs. healthy cardiomyocytes performed with Metascape. (**d**) Gene ontology (GO) term analysis for the 527 significantly up-regulated genes in hypertrophied vs. healthy cardiomyocytes performed with Metascape.

We selected the four cardiomyocyte clusters with 45,728 nuclei (of which 28,001 were from healthy controls and 17,727 were from patients with severe AS and hypertrophy) to address regulation of gene expression in hypertrophic compared to healthy control hearts. In total, 527 genes were significantly upregulated and 2,775 genes were significantly downregulated in hypertrophied cardiomyocytes (top downregulated and upregulated genes are visualized in a heatmap in Extended Data Fig. 3a,b, respectively). Gene ontology (GO) term analysis of significantly downregulated genes is shown in Extended Data Fig. 3c and that of upregulated genes is shown in Extended Data Fig. 3d. The analysis in Extended Data Fig. 3c revealed regulation of actin cytoskeleton organization, signaling by receptor tyrosine kinases and response to hormones. In detail, the relative proportion of MYH isoforms was significantly altered, with a higher expression of the gene *MYH7* encoding the slower isoform, whereas *MYH6*, which encodes the fast MYH isoform, was repressed in diseased patient hearts (Fig. 1b). Various other cardiomyocyte disease and stress markers (*NPPA*, *NPPB*, *MYH7*, *MYH7B*, *XIRP2*, *CMYA5*, *ANKRD1*, *TNNI3*, *ACTA1* and *PFKP)*, summarized as a cardiomyocyte disease score, showed significantly higher expression in hypertrophied cardiomyocytes (Fig. 1c).

**Extended Data Fig. 4:**
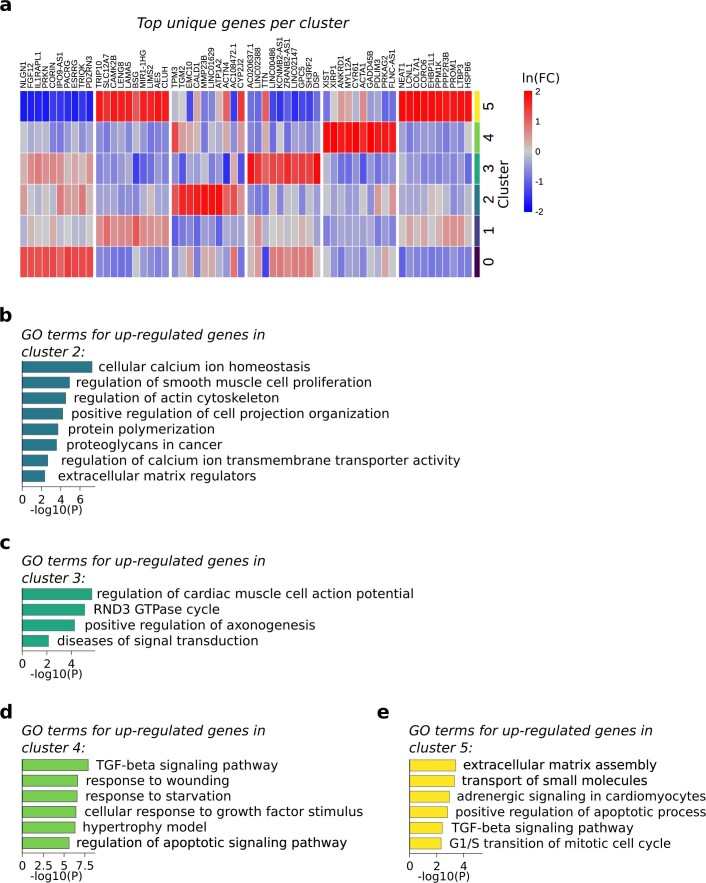
Cardiomyocyte sub-cluster specific signatures and gene ontology terms. (**a**) Heatmap for the top ten uniquely up-regulated genes (natural logarithm of the fold change) per cardiomyocyte sub-cluster. (**b-e**) Gene ontology (GO) term analysis for the genes up-regulated in the distinct cardiomyocyte sub-clusters vs. all other cardiomyocytes performed with Metascape. Adjusted p-values based on Bonferroni correction using all genes of the dataset to compare the expression in violin plots were calculated with the Seurat function ‘FindAllMarkers’ using ‘bimod’ as the statistical test (a).

To gain further detailed insights into the effect of hypertrophy on cardiomyocyte heterogeneity, we included all four cardiomyocyte clusters and performed a detailed subclustering analysis, resulting in six distinct cardiomyocyte clusters with unique gene expression profiles (Fig. 1d and Extended Data Fig. 4a).

**Extended Data Fig. 5:**
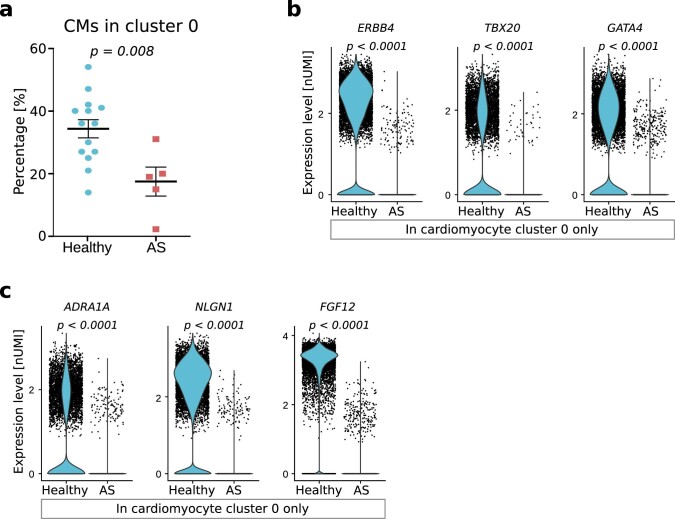
Cardiomyocyte sub-cluster 0 specific signatures. (**a**) Cardiomyocyte representation in cluster CM0 as percentage of all cardiomyocytes shown for each individual patient (Healthy n=14; AS n=5). (**b**) Violin plots for the genes ERBB4, TBX20 and GATA4 in cardiomyocytes of cluster 0 only (log2 transformed and normalized UMI counts). (**c**) Violin plots for the genes ADRA1A, NLGN1 and FGF12 in cardiomyocytes of cluster 0 only (log2 transformed and normalized UMI counts). Data are shown as mean ± s.e.m. (a). Normal distribution was assessed using the Kolmogorov-Smirnov test (a). Statistical analysis to compare two Gaussian distributed groups was performed using the unpaired, two-sided Student’s t-test (a). t=3,013, 17 degrees of freedom (a). Adjusted p-values based on Bonferroni correction using all genes of the dataset to compare the expression in violin plots were calculated with the Seurat function ‘FindAllMarkers’ using ‘bimod’ as the statistical test (b-c). Source data

Analysis of differential gene expression in cluster 0 showed evidence for imbalanced regulation of genes associated with muscle cell proliferation and development but also of those associated with blood circulation and synapse organization (Fig. 1e). Among the other cardiomyocyte subclusters, cluster 2 revealed a potential role in enhanced interactions with the extracellular matrix and actin binding (Extended Data Fig. 4a,b). Cluster 3 was characterized by potential involvement in the conduction system (Extended Data Fig. 4a,c), while cluster 4 demonstrated increased signatures of genes associated with cardiomyocyte stress and hypertrophy (Extended Data Fig. 4a,d). Cluster 5, which shared several signatures with cluster 1, revealed GO terms involved in extracellular matrix assembly, adrenergic signaling and apoptosis (Extended Data Fig. 4a,e). Of the six subclusters, only cluster 0 (CM0) was significantly reduced in hypertrophic hearts (Fig. 1f and Extended Data Fig. 5a). Genes that had significantly reduced expression in the hypertrophied human heart in cluster 0 included *ERBB4* (Erb-B2 receptor tyrosine kinase 4), *TBX20* (T-box transcription factor 20) and *GATA4* (GATA-binding protein 4) (Extended Data Fig. 5b). The products of *ERBB4* and *TBX20* are known to promote cardiomyocyte proliferation and regeneration^15,16^. These genes also had significantly reduced expression when analyzing the total cardiomyocyte population (Fig. 1g). *GATA4*, the product of which promotes regenerative responses after pressure overload^17^ but also controls expression of pro-angiogenic factors in cardiomyocytes^18^, was significantly downregulated in cardiomyocytes of the hypertrophied human myocardium (Fig. 1g and Extended Data Fig. 5b). In addition, an underexplored member of the fibroblast growth factor (FGF) family, *FGF12*, which was suggested as a candidate Brugada syndrome locus^19^, was significantly repressed in hypertrophic cardiomyocytes (Fig. 1g and Extended Data Fig. 5c).

Other interesting genes that were downregulated in the hypertrophic heart and linked to the GO term ‘synapse organization’ and ‘trans-synaptic signaling’ were *NLGN1* (neuroligin-1, which is critical for the formation and consolidation of synaptic connectivity but is also involved in vascular development and vessel maturation^20^) and *ADRA1A* (α-1A adrenergic receptor, which mediates cardioprotective signaling in the mouse and human heart^21^) (Fig. 1g and Extended Data Fig. 5c).

**Extended Data Fig. 6:**
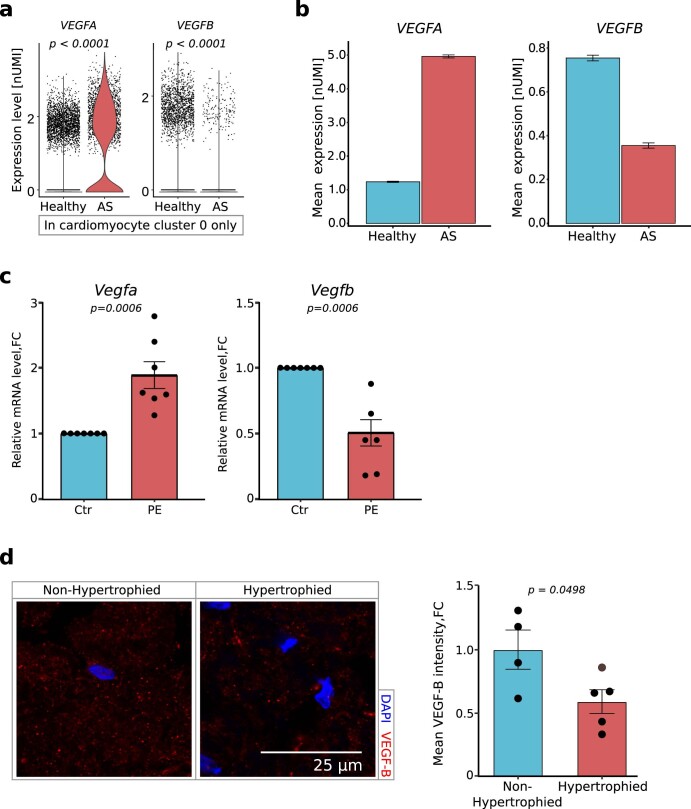
VEGFA-VEGFB switch in hypertrophied cardiomyocytes. (**a**) Violin plots for the genes VEGFA and VEGFB in cardiomyocytes of cluster 0 only (log2 transformed and normalized UMI counts). (**b**) Mean expression of VEGFA and VEGFB in healthy (n=14) and hypertrophied (AS, n=5) hearts (log2 transformed and normalized UMI counts). (**c**) Vegfa and Vegfb mRNA levels for untreated vs. phenylephrine (PE, 200μM, 72h) treated primary neonatal rat cardiomyocytes identified by qRT-PCR (houskeeping gene: Hprt, n=7, displayed as fold change to untreated cardiomyocytes). (**d**) Left: representative immunofluorescence images of cardiac cryosections from non-hypertrophied hearts and hearts from patients with hypertrophic cardiomyopathy. Blue: DAPI, red: VEGF-B. Scale bar indicates 25 μm. Right: quantification for histological assessment of VEGF-B protein expression in n=4 non-hypertrophied vs. n=5 hypertrophied hearts. Adjusted p-values based on Bonferroni correction using all genes of the dataset to compare the expression in violin plots were calculated with the Seurat function ‘FindAllMarkers’ using ‘bimod’ as the statistical test (a). Data are shown as mean ± s.e.m. (b-d). Normal distribution was assessed using the Kolmogorov-Smirnov test (c-d). t=2,368, seven degrees of freedom (d). Statistical analysis was performed using the Wilcoxon rank-sum test (c). Statistical analysis to compare two Gaussian distributed groups was performed using the unpaired, two-sided Student’s t-test (d). Source data

Among the genes related to the GO term ‘blood circulation’, we additionally found the two genes *VEGFA* and *VEGFB* to be dysregulated. *VEGFA*, the product of which is pro-angiogenic but also can induce vascular inflammation and leakage^22^, was highly upregulated in cardiomyocytes of the disease group in cluster 0 only (Extended Data Fig. 6a) as well as in the total cardiomyocyte population (Fig. 1h,i and Extended Data Fig. 6b). By contrast, *VEGFB*, the product of which does not regulate angiogenesis but can support vessel development, maturation and cardiac regeneration^23^, was significantly downregulated in both cluster 0 (Extended Data Fig. 6a) and the whole population (Fig. 1h,i and Extended Data Fig. 6b). This observation is consistent with increased *Vegfa* expression but downregulation of *Vegfb* mRNA expression in neonatal rat cardiomyocytes that were stimulated with phenylephrine (PE) to induce hypertrophy (Extended Data Fig. 6c). This finding is consistent with published studies showing downregulation of *Vegfb* mRNA in mouse hearts after pressure overload^24^. We further confirmed downregulation of VEGFB at the protein level in human hypertrophied heart sections compared to non-hypertrophied controls (Extended Data Fig. 6d; patient cohort characteristics are summarized in Supplementary Table 3). The imbalance of *Vegfa* and *Vegfb* expression may lead to a disconnect between sprouting angiogenesis and vessel maturation: while VEGFA can induce adaptive angiogenesis as observed in murine hearts after pressure overload^4^, low VEGFB levels may limit vessel maturation.

### Cellular communication in the hypertrophied heart

**Fig. 2:**
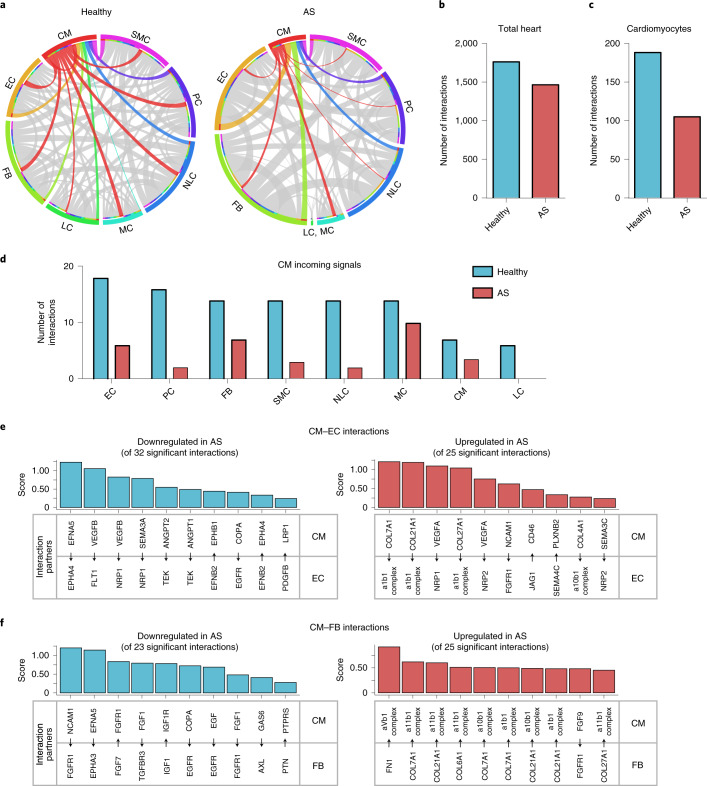
Disturbed intercellular crosstalk in hypertrophied hearts. **a**, Circos plots visualizing ligand–receptor interactions. Interactions between the different cell types were analyzed using CellPhoneDB to analyze cells from healthy hearts (left) and patients with AS (right). Incoming signals are shown in red. Outgoing signals are shown in the colors assigned to the respective cell type. The thickness of the lines indicates the number of interactions. **b**, Total ligand–receptor interactions in hearts of healthy individuals versus patients with AS. **c**, Total ligand–receptor interactions in cardiomyocytes of hearts of healthy individuals versus those with AS. **d**, Cardiomyocyte ligand–receptor interactions with other cardiac cell types. Shown are incoming signals (number of interactions) with a ligand expressed by any other cell type and the respective receptor expressed by cardiomyocytes. **e**,**f**, Representative cardiomyocyte ligand–receptor interaction scores calculated with CellPhoneDB are shown. **e**, Downregulated pathways from cardiomyocyte interactions with endothelial cells in hearts of patients with AS (left, of 32 total significant interactions) and upregulated pathways from those in hearts of patients with AS (right, of 25 total significant interactions). Direction of communication is indicated by arrows. **f**, Downregulated pathways from cardiomyocyte interactions with fibroblasts in hearts of patients with AS (left, of 23 total significant interactions) and upregulated pathways from those in hearts of patients with AS (right, of 25 total significant interactions). ANGPT, angiopoietin; COL, collagen; EGFR, epidermal growth factor receptor; FGFR1, FGF receptor 1; FN1, fibronectin 1; GAS6, growth arrest-specific 6; IGF1, insulin-like growth factor 1; IGF1R, IGF1 receptor; JAG1, jagged canonical Notch ligand 1; LRP1, LDL receptor-related protein 1; NCAM1, neural cell adhesion molecule 1; NRP2, neuropilin 2; PLXNB2, plexin B2; PTN, pleiotrophin; PTPRS, protein tyrosine phosphatase receptor type S; SEMA, semaphorin; TGFBR3, transforming growth factor β receptor 3. Statistical analysis for interaction pathways was assessed using the CellPhoneDB package in R (**e**,**f**).

**Extended Data Fig. 7:**
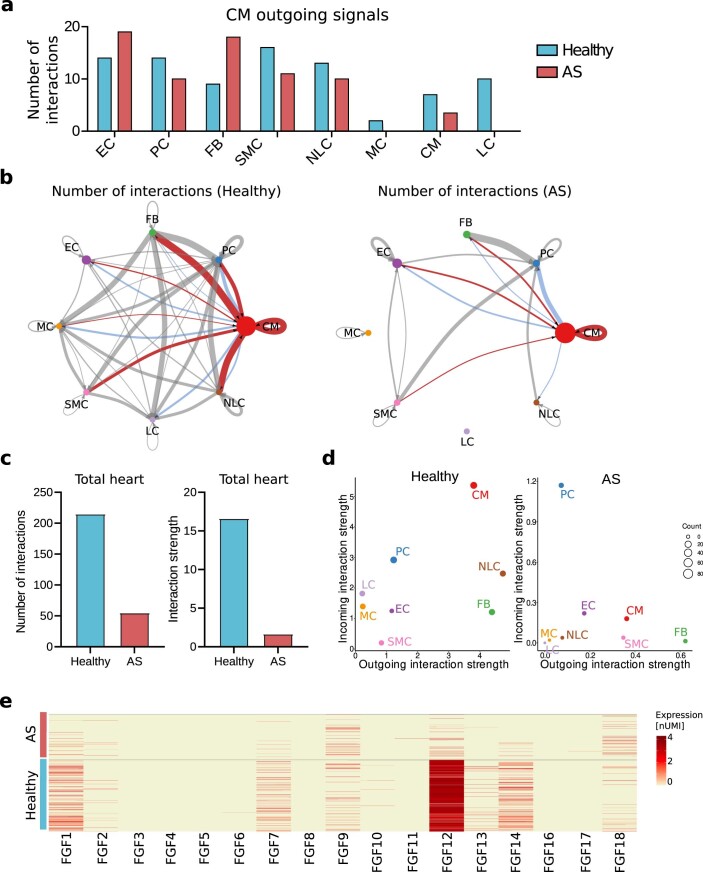
Cross-talk between cardiomyocytes and other cardiac cells in hypertrophy. (**a**) Cardiomyocyte (=CM) ligand-receptor interactions with other cardiac cell-types in the healthy (blue) or aortic valve stenosis (=AS) heart (red). Shown are outgoing signals (number of interactions) from CMs to the respective interacting gene expressed by any other cell type. PC=pericytes; FB=fibroblasts; EC=endothelial cells; MC=monocytes; SMC= smooth muscle cells; LC=leukocytes; NLC=neuronal-like cells. (**b**) Circos plots visualizing the number of ligand receptor interactions analyzed using CellChat between the different cell types in cells from healthy hearts (left panel) and aortic valve stenosis (=AS) patients (right panel). Cardiomyocyte incoming signals are shown in red, outgoing signals are shown in blue. (**c**) Left: Total number of ligand-receptor interactions in hearts of healthy vs. AS patients assessed by using CellChat. Right: Total strength of ligand-receptor interactions in hearts of healthy vs. AS patients assessed with CellChat. (**d**) Dot plots for the number and strength of incoming and outgoing interactions for all cell types in healthy (left) vs. AS patients (right) assessed with CellChat. (**e**) Heatmap for genes from the fibroblast growth factor (FGF) family expressed in CMs (log2 transformed and normalized UMI counts) in healthy hearts vs. hearts with AS. Color refers to the expression.

Next, we questioned whether cellular communication between cells, particularly between cardiomyocytes and endothelial cells, may be compromised in the hypertrophic heart. In silico analysis of ligands and receptors in individual cells allows for the prediction of cell–cell communication via ligand–receptor complexes^25^. Analysis of our data with the bioinformatic tool CellPhoneDB^25^ revealed various changes in cellular interactions in the healthy versus the hypertrophic heart (Fig. 2). Overall, a reduction in cellular interactions was noted when assessing all interactions (Fig. 2a,b). In particular, cardiomyocyte interactions were reduced in the hypertrophic heart (Fig. 2a,c). This decrease in interactions was predominantly caused by a profound reduction in incoming signals to cardiomyocytes from most cell types as shown by the Circos plot (Fig. 2a; reduced interactions with cardiomyocytes are indicated by thinning of red-labeled connections) and by quantitative analysis (Fig. 2c,d). Of note, cardiomyocyte outgoing signals showed a different pattern, with increased outgoing signals to endothelial cells and fibroblasts, while signals to other mural cells such as pericytes or smooth muscle cells appeared to be lesser (Fig. 2a and Extended Data Fig. 7a). Similar findings were shown when using the recently developed bioinformatic tool CellChat^26^ to predict cellular interactions (Extended Data Fig. 7b–d). Again, cardiomyocyte communication with other cell populations was diminished (Extended Data Fig. 7b–d). While cardiomyocytes received most outgoing and incoming signals in the healthy heart, this was not the case in the hypertrophic heart (Extended Data Fig. 7d).

Among the top regulated interactions between cardiomyocytes and endothelial cells, we specifically noted alterations in VEGF and ephrin ligand interactions with their respective receptors (Fig. 2e). Reduced Eph–ephrin interactions were predicted for the cardiomyocyte-expressed ligand EFNA5 with endothelial ephrin (EPH)A4 (Fig. 2e) and for endothelial-derived EFNB2 interactions with the cardiomyocyte-expressed receptors EPHB1 and EPHA4 (Fig. 2e). Moreover, reduced interaction of cardiomyocyte-derived VEGFB with its receptors FLT1 and NRP1 was noted, whereas VEGFA interactions were increased in disease states (Fig. 2e). The latter is in accordance with the regulation of VEGFA and VEGFB shown above (Fig. 1h,i) and with published studies showing important roles of VEGFA and VEGFB in cardiomyocyte–endothelial crosstalk^4,27^.

Disturbed communication also affected interactions of cardiomyocytes and fibroblasts. As expected, interactions of various fibroblast-derived collagens were augmented in the diseased heart (Fig. 2f, right). Interestingly, in silico analysis predicted further alterations in FGF–receptor interactions, with a downregulation of FGF1 interactions but increased FGF9 communication from cardiomyocytes to fibroblasts in disease states (Fig. 2f and Extended Data Fig. 7e). Overall, these data suggest a model in which cardiomyocyte communication with endothelial cells and fibroblasts is critically disturbed in the diseased heart.

### Disturbed Eph receptor interactions in cardiac hypertrophy

**Fig. 3:**
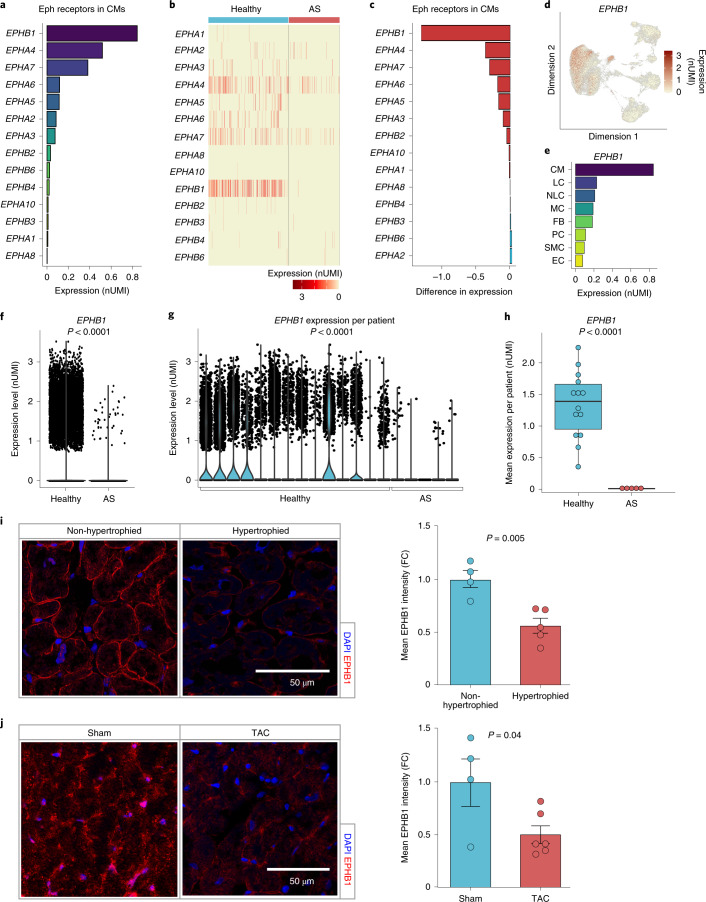
*EPHB1* is dysregulated in cardiomyocytes of hypertrophied hearts. **a**, Mean expression (log_2_-transformed and normalized UMI counts) of Eph receptors in all cardiomyocytes. **b**, Heatmap of Eph receptors expressed (log_2_-transformed and normalized UMI counts) in all cardiomyocytes of healthy individuals versus patients with AS. Colors indicate expression levels in each nucleus. **c**, Differential expression of Eph receptors in cardiomyocytes between hearts of healthy individuals versus patients with AS, ranked by differential expression and calculated by subtracting mean expression of genes (normalized UMI counts; values from individuals with hypertrophy minus those from healthy individuals). **d**, FeaturePlot for expression of *EPHB1* (log_2_-transformed and normalized UMI counts). Color reflects expression levels in each nucleus. **e**, *EPHB1* expression (log_2_-transformed and normalized UMI counts) according to cell type. **f**, Violin plot for *EPHB1* expression in cardiomyocytes (log_2_-transformed and normalized UMI counts). **g**, Violin plot for *EPHB1* expression in cardiomyocytes from patients with AS versus healthy individuals shown for individual patients (log_2_-transformed and normalized UMI counts). **h**, Box plot showing expression of *EPHB1* in cardiomyocytes in individual patients (healthy, *n* = 14; AS, *n* = 5; log_2_-transformed and normalized UMI counts, visualized as median with 25th and 75th percentiles, with whiskers indicating maximal and minimal values). **i**, Left, representative immunofluorescence images of cryosections from non-hypertrophied hearts and hearts from patients with hypertrophic cardiomyopathy. Blue, 4,6-diamidino-2-phenylindole (DAPI); red, EPHB1. Scale bar, 50 µm. Right, quantification for histological assessment of EPHB1 protein expression in *n* = 4 non-hypertrophied versus *n* = 5 hypertrophied hearts. FC, fold change. **j**, Left, representative immunofluorescence images of cardiac cryosections from sham-operated mice and operated mice in the TAC model. Blue, DAPI; red, EPHB1. Scale bar, 50 µm. Right, quantification for histological assessment of EPHB1 protein expression in *n* = 4 hearts of sham-operated mice versus *n* = 6 hearts of mice in the TAC model. Adjusted *P* values based on Bonferroni correction using all genes in the dataset to compare expression in violin plots were calculated with the Seurat function ‘FindAllMarkers’ using ‘bimod’ as the statistical test (**f**,**g**). Data are shown as mean ± s.e.m. (**i**,**j**). Normal distribution was assessed using the Kolmogorov–Smirnov test (**h**–**j**). Statistical analysis to compare two groups was performed using unpaired, two-sided Student’s *t*-tests (**h**–**j**). *t* = 4,118, seven degrees of freedom (**i**); *t* = 2,477, eight degrees of freedom (**j**). Source data

**Extended Data Fig. 8:**
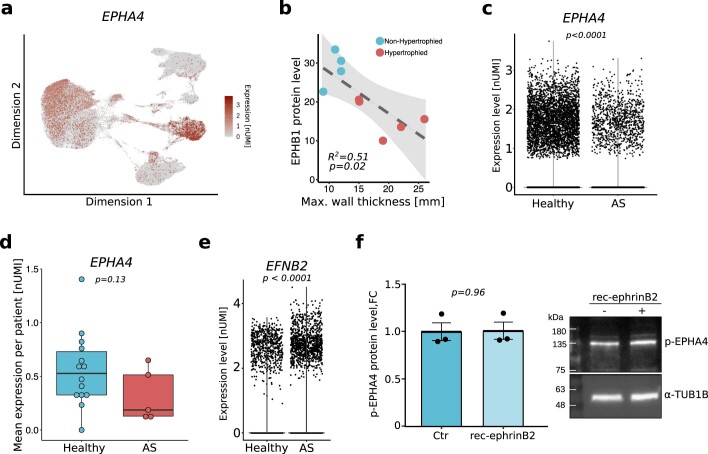
Regulation of the potential ephrin-B2 receptor EPHA4. (**a**) FeaturePlot for the expression of EPHA4 in snRNA-seq dataset (log2 transformed and normalized UMI counts). Color refers to the expression. (**b**) Linear regression for EPHB1 protein expression in cardiomyocytes (see Fig. 4i) and maximal wall thickness in mm (see Supplementary Data 3), shown for individual patients (Healthy n=14; AS n=5). The confidence bands reflect the standard error of the beta coefficients. (**c**) Violin plot for EPHA4 in hypertrophied (AS) vs. healthy cardiomyocytes (log2 transformed and normalized UMI counts). (**d**) Box plot showing expression of EPHA4 in cardiomyocytes in individual patients of respective groups (n=14 healthy, n=5 AS) (log2 transformed and normalized UMI counts; visualized as median and 25th and 75^th^ percentiles with whiskers indicating maximal and minimal values). (**e**) Violin plot for EFNB2 in hypertrophied (AS) vs. healthy cardiomyocytes (log2 transformed and normalized UMI counts). (**f**) Phosphorylated EPHA4 protein in human cardiomyocytes treated with recombinant ephrin-B2 (10μg/ml, 15min), compared to non-treated CMs (=Ctr). Left: Quantification displayed as fold change vs Ctr (n=3; normalized to α-Tubulin-1B); Right: representative Western blot. α-Tubulin-1B served as loading control. Blots were processed in parallel and the loading control run on the same blot. Adjusted p-values based on Bonferroni correction using all genes of the dataset to compare the expression in violin plots were calculated with the Seurat function ‘FindAllMarkers’ using ‘bimod’ as the statistical test (c, e). Data are shown as mean ± s.e.m. (f). Normal distribution was assessed using the Kolmogorov-Smirnov test (d, f). t=0,05916, four degrees of freedom (f). For Gaussian distributed data, statistical analysis to compare two groups was performed using the unpaired, two-sided Student’s t-test (d, f). Source data

Because analysis of differentially expressed genes and in silico prediction of cellular interactions suggest a profound alteration of endothelial–cardiomyocyte crosstalk in the human hypertrophied heart, we next evaluated the functional impact of these findings. We focused on exploring the role of Eph–ephrin interactions, which have not been studied thus far. To gain insights into the possible importance of regulated Eph–ephrin interactions, we first determined the expression of Eph receptors in cardiomyocytes (Fig. 3a–c). This analysis revealed that *EPHB1* was highly expressed and enriched in cardiomyocytes (Fig. 3a–e), whereas *EPHA4* was highly expressed in cardiomyocytes but was also detected in other cell populations (Extended Data Fig. 8a). Many members of the EPHA and EPHB subclasses were downregulated in cardiomyocytes of hypertrophic hearts (Fig. 3b,c). Most prominently, *EPHB1* expression was almost absent in the hypertrophic heart (Fig. 3b,c). Violin plots confirmed the significant downregulation of *EPHB1* when analyzing total cardiomyocytes of healthy individuals versus patients with hypertrophy (Fig. 3f). Of note, individual violin plots per individual confirmed consistently low expression of *EPHB1* in patients with hypertrophy compared to healthy controls (Fig. 3g). Significant downregulation was also documented when the mean of *EPHB1* expression was calculated for each patient to compare healthy individuals to those with disease (Fig. 3h). Moreover, histological analysis confirmed that cardiomyocyte EPHB1 protein expression was significantly reduced in patients with hypertrophic cardiomyopathy (Fig. 3i; patient cohort characteristics are summarized in Supplementary Table 3). Likewise, EPHB1 protein levels were significantly reduced in hypertrophied hearts of mice exposed to transverse aortic constriction (TAC)-induced pressure overload (Fig. 3j). EPHB1 protein levels were inversely correlated with maximum heart wall thickness, with the lowest levels being found in hypertrophied hearts with thickened walls (Extended Data Fig. 8b).

*EPHA4* was also significantly repressed when analyzing total cells (Extended Data Fig. 8c); however, when mean values of all cardiomyocytes per patient were used, no significant difference was shown between hypertrophic and healthy control groups (Extended Data Fig. 8d).

**Fig. 4:**
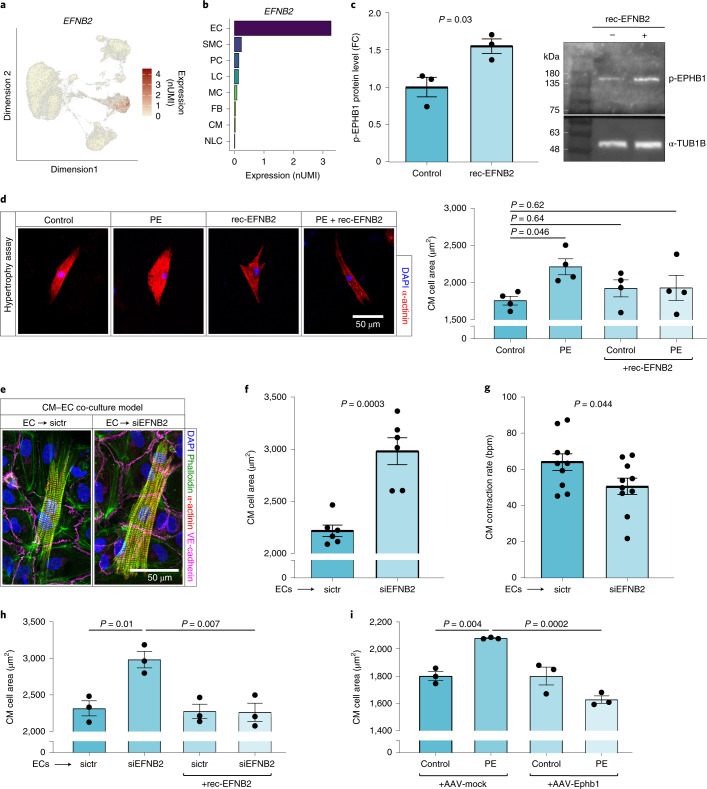
The EFNB2–EPHB1 ligand–receptor interaction has anti-hypertrophic effects in cardiomyocytes. **a**, FeaturePlot of *EFNB2* expression in the human cardiac snRNA-seq dataset (log_2_-transformed and normalized UMI counts). Color reflects expression levels in each nucleus. **b**, Mean *EFNB2* expression (log_2_-transformed and normalized UMI counts) per cell type. **c**, Phosphorylated (p) EPHB1 protein levels in human cardiomyocytes treated with recombinant (rec)-EFNB2 (15 min) compared to those in untreated cardiomyocytes (control). Left, quantification as fold change versus control (*n* = 3; normalized to α-tubulin-1B (α-TUB1B). Right, representative western blot. α-tubulin-1B served as the loading control. Blots were processed in parallel; the loading control was run on the same blot. **d**, Left, representative immunofluorescence images of the hypertrophy assay with primary neonatal rat cardiomyocytes (PE, recombinant EFNB2, 72 h). Blue, DAPI; red, sarcomeric α-actinin. Right, quantification of the hypertrophy assay only counting individual cardiomyocytes. The cardiomyocyte cellular area is displayed in µm^2^ (*n* = 4). **e**, Representative immunofluorescence images of the co-culture model with primary neonatal rat cardiomyocytes and endothelial cells (**e**–**i**). Endothelial cells were treated with a small interfering (si)RNA pool targeting *EFNB2* (siEFNB2) or a non-targeting siRNA pool (sictr) before seeding cardiomyocytes (**e**–**h**). Blue, DAPI; green, phalloidin; red, sarcomeric α-actinin; violet, VE-cadherin. **f**, Quantification of cardiomyocyte cellular area after endothelial treatment with sictr and siEFNB2 (*n* = 6; µm^2^). **g**, Quantification of the cardiomyocyte contraction rate shown in beats per min (bpm) after endothelial pretreatment with sictr and siEFNB2 (*n* = 10). **h**, Quantification of cardiomyocyte cellular area after endothelial pretreatment with sictr and siEFNB2 and additional treatment of co-cultured cells with recombinant EFNB2 protein (*n* = 3, µm^2^). **i**, Quantification of cardiomyocyte cellular area after EPHB1 overexpression, mediated by AAV6 (AAV-mock, AAV-Ephb1) using a PE-mediated hypertrophy model (72 h, *n* = 3). Data are shown as mean ± s.e.m. (**c**,**d**,**f**–**i**). Normal distribution was assessed using the Shapiro–Wilk test (**c**) or the Kolmogorov–Smirnov test (**d**,**f**–**i**). Statistical analysis for comparison of two groups was performed using unpaired, two-sided Student’s *t*-tests (**c**,**f**,**g**). *t* = 3,400, four degrees of freedom (**c**); *t* = 5,509, ten degrees of freedom (**f**); *t* = 2,170, 18 degrees of freedom (**g**). Statistical analysis was performed using one-way ANOVA with post hoc Tukey tests for data with a Gaussian distribution (**h**,**i**) and one-way ANOVA with Dunnett’s correction for pairwise comparisons (**d**). Source data

It has been shown that EPHB1 and EPHA4 can be activated by the ligand EFNB2 in neural crest cells^28^. *EFNB2* is highly expressed and enriched in endothelial cells compared to other cells of the heart (Fig. 4a,b and Extended Data Fig. 8e). These expression patterns could imply that endothelial EFNB2 stimulates cardiomyocyte EPHB1 and EPHA4 receptors in the healthy heart, whereas reduced expression of these Eph receptors prevents this interaction in the hypertrophic heart. As the effects of EFNB2 on Eph receptor stimulation in cardiomyocytes have not been investigated previously, we first determined the effect of recombinant EFNB2 on EPHB1 and EPHA4 phosphorylation in human cardiomyocytes. We confirmed an increase in phosphorylation of EPHB1 upon EFNB2 stimulation (Fig. 4c), whereas this was not the case for EPHA4 (Extended Data Fig. 8f), suggesting that the interaction with EPHB1 is more relevant.

**Extended Data Fig. 9:**
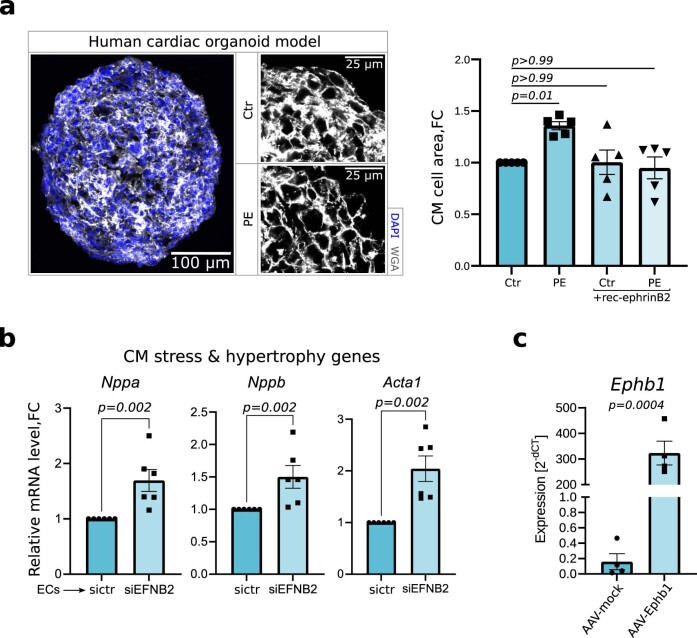
Anti-hypertrophic potential of the EPHB1-EFNB2 interaction. (**a**) Human multicellular cardiac organoid model. Quantification and representative immunofluorescence images for the hypertrophy assay analyzing cardiomyocytes in the cardiac organoids (PE=phenylephrine, 200μM, 72h; recombinant (rec) ephrin-B2 protein, 10μg/ml, 72h). Blue: DAPI, white: WGA. Scale bar: 100μm or 25μm. The cardiomyocyte cellular area is displayed as fold change to Ctr (n=5). (**b**) CM stress and hypertrophy markers *Nppa*, *Nppb* and *Acta1* mRNA levels in primary rat neonatal cardiomyocytes after endothelial treatment with sictr and siEFNB2 (housekeeping gene: *Hprt*, n=6, displayed as fold change to sictr). (**c**) *Ephb1* mRNA levels in neonatal rat cardiomyocytes after treatment with adeno-associated virus type 6 (AAV-mock; AAV-Ephb1; housekeeping gene: *Hprt*, n=4, displayed as 2-ΔCt). Data are shown as mean ± s.e.m. (a-c). Normal distribution was assessed using the Kolmogorov-Smirnov test (a-c). Statistical analysis for data without Gaussian distribution to compare two groups was performed using the Wilcoxon rank-sum test (b). For Gaussian distributed data, statistical analysis to compare two groups was performed using the unpaired, two-sided Student’s t-test (c). t=6,967, six degrees of freedom (c). For more than two groups, statistical analysis was performed using the Kruskal-Wallis test with a post hoc Dunn test for data without Gaussian distribution (a). Source data

Downregulation of *EPHB1* in cardiac hypertrophy would imply that EFNB2–EPHB1 interactions might inhibit the hypertrophic response. Therefore, we investigated whether EFNB2 may interfere with cardiomyocyte hypertrophy. Indeed, stimulation with exogenous recombinant EFNB2 protected neonatal rat cardiomyocytes from PE-induced hypertrophy in vitro (Fig. 4d). The anti-hypertrophic effect of EFNB2 was further confirmed in a more physiological multicellular cardiac tissue mimetic model (Extended Data Fig. 9a).

To further investigate whether endothelial-derived EFNB2 might mediate an anti-hypertrophic effect on cardiomyocytes via activation of EPHB1, we established a co-culture model in which cardiomyocytes were plated on top of cultured human umbilical cord endothelial cells (Fig. 4e,f). Interestingly, silencing of the gene encoding the ligand EFNB2 in endothelial cells induced hypertrophy in cardiomyocytes as demonstrated by increased cardiomyocyte size (Fig. 4e,f). *EFNB2* silencing in endothelial cells further reduced the cardiomyocyte contraction rate (Fig. 4g) and augmented cardiac stress markers (Extended Data Fig. 9b) in co-cultured cardiomyocytes. Importantly, the addition of exogenous recombinant EFBN2 rescued the failed anti-hypertrophic response in co-cultures of *EFNB2*-silenced endothelial cells and cardiomyocytes (Fig. 4h), supporting the causal and direct involvement of EFNB2 in the observed cellular communication.

To confirm the functional role of EPHB1, we used adeno-associated virus type 6 (AAV6) transduction to augment *EPHB1* expression in cardiomyocytes (Extended Data Fig. 9c). Indeed, overexpression of EPHB1 significantly prevented PE-induced hypertrophy of cardiomyocytes in the co-culture system (Fig. 4i). In sum, these data indicate a protective function of the heterocellular EFNB2–EPHB1 interaction between cardiomyocytes and endothelial cells.

## Discussion

In summary, the present study reveals a profound dysregulation of genes in cardiomyocytes of hypertrophied hearts of patients suffering from AS, of which many are involved in regulating the interaction of cardiomyocytes with other non-parenchymal cells. In particular, genes involved in communication with endothelial cells were strikingly regulated. Among these, genes encoding Eph receptors and ephrin ligands stand out.

In detail, we identified extensive dysregulation of the Eph receptor EPHB1 in human cardiac hypertrophy, highlighting the importance of this pathway for future therapeutic interventions. Both activation of EPHB1 with recombinant EFNB2 and overexpression of the receptor EPHB1 prevented PE-induced hypertrophy of cardiomyocytes in vitro. A few previous studies reported on functions of other Eph receptors in cardiomyocytes: *EPHA4* was found to be highly expressed in the atria and its deletion leads to electrocardiographic abnormalities^29^, whereas EPHB4 was shown to regulate cardiac progenitor cell differentiation^30^. However, the role of EPHB1 in cardiomyocytes has not been previously addressed. Our findings suggest that EFNB2 interaction with EPHB1 regulates hypertrophy but additionally influences cardiomyocyte contraction rate and stress responses. Of note, although we confirmed the effects of EPHB1 in a human multicellular organoid context, its role in disease pathologies in vivo and the mechanism of action are unclear. From studies with other cell types, it is known that EPHB1 activates various signaling pathways involved in cell migration, proliferation and differentiation but can also crosstalk with other receptor tyrosine kinases^31,32^. Other ephrins, such as the ligand EFNB1, were shown to interact with the claudin-5–ZO-1 complex at the lateral membrane in cardiomyocytes, which is essential for sarcomeric and intercalated disk structural disorganization^33^. Given the complex activities of ephrins, further studies are required to elucidate downstream signaling of EPHB1 in cardiomyocytes.

Interestingly, our data demonstrate that silencing of *EFNB2* in endothelial cells is sufficient to induce cardiomyocyte hypertrophy and the stress response in endothelial–cardiomyocyte co-culture models. These data suggest that continuous EFNB2 expression in endothelial cells is required to maintain cardiomyocyte homeostasis. The overall role of EFNB2 is complex as it can act not only on cardiomyocytes and other cells but also has cell-autonomous functions in endothelial cells. Deletion of *Efnb2* leads to defects in angiogenesis and myocardial trabeculation, resulting in embryonic lethality^32^. In adult mice, EFNB2–Fc increased endothelial cell proliferation and angiogenesis and induced neovascularization after myocardial infarction^34^, whereas genetic deletion of *Efnb2* or interference with EFNB2 reverse signaling induced microvascular destabilization and increased fibrosis^35,36^. Overall, these studies suggest an important role of EFNB2 in vessel growth and stabilization. However, other studies demonstrate that suppressing *Efnb2* expression by lentiviruses carrying *Efnb*2 short hairpin RNA ameliorated cardiac fibrosis and improved cardiac function in a mouse model of myocardial infarction^37^. Thus, EFNB2 appears to exhibit a double-edged role in cardiac injury responses. One may speculate that the increase in endothelial *EFNB2* expression observed in endothelial cells of patients with hypertrophic hearts might induce angiogenesis and protect cardiomyocytes at the expense of inducing a pro-fibrotic response. It will be important to determine the time course of these cellular interactions to distinguish to what extent the dysregulated cellular interactions causally contribute to cardiac hypertrophy.

Disturbed intercellular communication in human cardiac hypertrophy exceeds Eph–ephrin crosstalk and includes various other connections. The profoundly dysregulated expression of VEGF family members and other angiogenesis modulators in cardiomyocytes of the hypertrophied heart is likely to affect the function and maturation of the vascular network. In particular, increased expression of *VEGFA* may translate into augmentation of capillary growth in response to pressure overload as also shown in the mouse heart^4^. However, the simultaneous reduction in *VEGFB* expression may limit vessel maturation, resulting in immature capillary formation. In addition, VEGFB controls fatty acid uptake by endothelial cells^38^. Downregulation of *VEGFB* in the human hypertrophied heart may therefore result in an insufficient uptake of fatty acids and reduce access to fatty acids, limiting cardiac metabolism.

### Limitations

**Extended Data Fig. 10:**
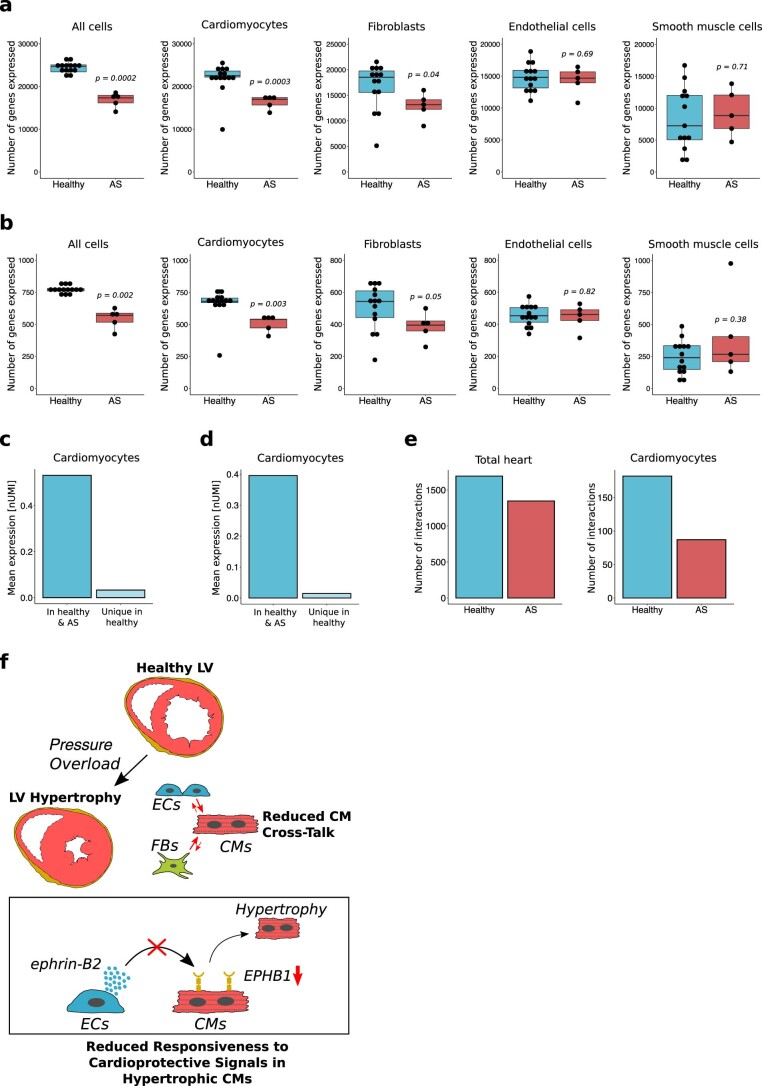
Cell-type specific number of genes. (**a**) Box plots for the number of genes expressed in cells from healthy (n=14) and hypertrophied (n=5; AS) hearts shown for all cells, cardiomyocytes, fibroblasts, endothelial cells, and smooth muscle cells. Median with 25th and 75^th^ percentiles and whiskers indicating maximal and minimal values is shown. (**b**) As in (a) but filtered for genes included within the ligand-receptor analysis by CellphoneDB. Boxes with median and 25th and 75^th^ percentiles with whiskers indicating maximal and minimal values are shown. (**c**) The mean expression of shared genes expressed by cardiomyocytes from healthy and hypertrophied hearts and genes expressed uniquely by cardiomyocytes from healthy hearts (log2 transformed and normalized UMI counts). (**d**) As in (c) but filtered for genes included within the ligand-receptor analysis by CellphoneDB. (**e**) Ligand-receptor analysis was performed with CellphoneDB using only genes expressed by both, healthy and diseased (AS) hearts (therefore the underlying analysis is based on the same number of genes expressed between healthy and diseased hearts). Left: Total ligand-receptor interactions in healthy vs. hypertrophied hearts. Right: Total cardiomyocyte ligand-receptor interactions of healthy vs.hypertrophied hearts. (**f**) Cartoon visualizing the working hypothesis of reduced cardiomyocyte cross-talk in hypertrophied hearts and reduced responsiveness of cardiomyocytes to cardioprotective signaling (LV=left ventricle, ECs=endothelial cells, FBs=fibroblasts, CMs=cardiomyocytes). Normal distribution was assessed using the Kolmogorov-Smirnov test (a-b). Statistical analysis to compare two groups was performed using the unpaired, two-sided Student’s t-test (a-b).

As this study is based on the integration of several snRNA-seq datasets, we recognize limitations that arise due to differences in cell-capture efficiency, sequencing depth and library-preparation protocols from the different data sources and unintended biases from surgical sampling. Also, the number of genes was lower in diseased hearts in some cell types such as cardiomyocytes, which may have contributed to the reduction in ligand–receptor interactions in the disease state (Extended Data Fig. 10a,b). However, unique genes in the healthy heart were expressed at low levels (Extended Data Fig. 10c,d). Moreover, removing uniquely expressed genes in healthy heart samples followed by reanalysis of ligand–receptor interactions revealed similar results, namely, a reduction of ligand–receptor interaction in the diseased heart (Extended Data Fig. 10e). Furthermore, one cluster (cluster 8) was removed from downstream analysis based on an unbiased threshold value to avoid annotation of a cluster that may have resulted from sample-specific biases or restrictions of the integration method. Although we used state-of-the-art cell culture and organoid models, the potential therapeutic value of the findings needs to be verified in vivo.

In conclusion, the present data provide insights into the orchestrated network of intercellular circuits of communication that are impaired in the hypertrophic heart (see also the cartoon summarizing the findings in Extended Data Fig. 10f). Knowing that coordinated interactions are essential to maintain a healthy heart, interfering with the described pathways may provide therapeutic options. The high-quality dataset generated in the present study may additionally provide a valuable reference and tool for further deciphering cardiac diseases in humans. Pooling these data with snRNA-seq data from other sources using the bioinformatic approach taken in the present study may increase the power of the study and will allow molecular definition of different types of heart failure and decipherment of the impact of patient heterogeneity and comorbidities.

## Methods

### Human single-nucleus RNA-sequencing data

Two human snRNA-seq datasets were used: data from healthy cardiac tissue from the septum of 14 individuals in the Litvinukova et al. study^10^ and data from location-matched hypertrophic cardiac tissues from five patients with aortic stenosis^39^ (characteristics are summarized in Supplementary Tables 1 and 2). Biopsies were taken from five aortic stenosis samples during surgery of the aortic valve, with tissue from the hypertrophied interventricular septum in the left ventricle being collected (75–250 mg per sample; Department of Cardiovascular Surgery, University Hospital Frankfurt, Germany). Informed consent was obtained from all five patients. The study was approved by an institutional review committee of the University Hospital of the Johann Wolfgang Goethe University in compliance with internal standards of the German government, and procedures followed were in accordance with institutional guidelines (application 347/18) and the Declaration of Helsinki.

### Nuclear isolation

For patients with aortic stenosis, the following protocol was used: cardiac tissues were thawed on ice and cut into small pieces. Minced tissue was pre-digested with a 5-ml enzyme solution of collagenase (2,500 U, Thermo Fisher Scientific) in HBSS+/+ (Gibco) for 10 min at 37 °C in a water bath. After centrifugation at 500*g* and 4 °C for 5 min, the supernatant was discarded, and nuclei were isolated after cell disruption with a glass dounce homogenizer (five strokes with a loose pestle and ten strokes with a tight pestle)^40,41^. After filtering (20-µm strainer, pluriSelect), the suspension was centrifuged at 1,000*g* and 4 °C for 6 min and resuspended in 500 µl staining buffer containing 1% BSA (Sigma-Aldrich), 5 nM MgCl_2_ (Sigma-Aldrich), 1 mM EDTA (Gibco), 1 mM EGTA (Gibco), 0.2 U µl^−1^ RNasin Plus Inhibitor (Promega) and 0.1 µg ml^−1^ Hoechst (Life Technologies) in Dulbecco´s phosphaste buffered saline (DPBS). Hoechst-positive nuclei were separated from cell debris by using the FACSAria Fusion instrument (BD Biosciences) and sorted into staining buffer without Hoechst at 4 °C.

### Single-nucleus RNA-sequencing library preparation

Nuclear suspensions were loaded on a 10x Chromium Controller (10x Genomics) according to the manufacturer’s protocol. All snRNA-seq libraries were prepared using the Chromium Single Cell 3′ version 3 Reagent kit (10x Genomics) according to the manufacturer’s protocol. Individual nuclei were isolated into droplets together with gel beads coated with unique primers bearing 10x cell barcodes, UMIs and poly(dT) sequences. Reverse transcription reactions generated barcoded full-length cDNA followed by disruption of emulsions using the recovery agent and cDNA cleanup with DynaBeads MyOne Silane Beads (Thermo Fisher Scientific). Total cDNA was amplified using a Biometra Thermocycler TProfessional Basic Gradient with a 96-well sample block (98 °C for 3 min; cycled 14×: 98 °C for 15 s, 67 °C for 20 s and 72 °C for 1 min; 72 °C for 1 min; held at 4 °C). Amplified cDNA products were cleaned with the SPRIselect Reagent kit (Beckman Coulter). Indexed sequencing libraries were constructed using reagents from the Chromium Single Cell 3′ version 3 Reagent kit as follows: fragmentation, end repair and A tailing; size selection with SPRIselect; adaptor ligation; post-ligation cleanup with SPRIselect; sample index PCR and cleanup with SPRIselect beads. Library quantification and quality assessment were performed using the Bioanalyzer Agilent 2100 with a High Sensitivity DNA chip (Agilent Genomics). Indexed libraries were pooled equimolarly and sequenced on the Illumina NovaSeq 6000 by GenomeScan using paired-end 26 × 98-bp reads as the sequencing mode.

### Single-nucleus RNA-sequencing data integration and analysis

The two datasets were integrated using Harmony version 1.0 (ref. ^14^). To merge metadata vectors from all datasets (for example, UMI count, feature count, mitochondrial content or cell type), character strings indicating barcodes in the dataset of the metadata table were adapted and named properly. Before both datasets were integrated with Harmony, expression matrices were normalized and scaled with ‘NormalizeData()’, ‘FindVariableFeatures()’ and ‘ScaleData()’ using default settings. We used the two dataset names as covariates for the Harmony integration. Using ‘RunPCA()’, we obtained the reduced dimensionality, which is the input for the ‘RunHarmony()’ function, which starts the integration. To visualize the integration, we created a UMAP with ‘RunUMAP()’, for which we used the first 15 dimensions from the Harmony output. The resulting integrated dimensional embedding from Harmony was used for visualization, and graph embedded cells running on the SNN nearest-neighbor embedding were used afterward for clustering and cell type annotation. Local inverse Simpson’s index (LISI) was calculated in the local neighborhood of each nucleus by using the package LISI^14^. Neighborhoods populated only by one dataset are assigned an LISI score of 1, while neighborhoods represented equally by both datasets are assigned a score of 2. Potential doublets were detected using the package DoubletFinder (version 2.0.3)^42^.

Single-nucleus expression data were processed using the Cell Ranger Single Cell Software Suite (version 3)^43^. After harmonization, a total of 88,536 nuclei were used for further analysis. Unsupervised clustering was performed in Seurat (version 4.0.2) with a resolution of 0.25 using 12 dimensions following the Satija Lab Tutorial (https://satijalab.org/seurat/v3.1/pbmc3k_tutorial.html). To avoid artificial clusters that might arise from potential doublets or poor sample integration, we used the ‘identify_outlier()’ function from the package rstatix (version 0.6.0), identifying outliers based on the ratio of the number of genes and the LISI score. Mean ratios above Q3 + 1.5 × IQR or below Q1 − 1.5 × IQR were considered outliers and resulted in exclusion of the respective cluster (Q1, first quartile; Q3, third quartile; IQR, interquartile range).

Differential transcriptional profiles by cluster were generated in Seurat with associated GO terms derived from the databases selected in the ‘Express Analysis’ option from the functional annotation tool Metascape^44^. For receptor–ligand interactions and crosstalk analysis, CellPhoneDB^25^ or CellChat (version 1.1.43)^26^ were used, and interactions were illustrated using Circos^45^. We followed the standard tutorial ‘Comparison analysis of multiple datasets using CellChat’ from the CellChat GitHub repository (https://github.com/sqjin/CellChat).

### Neonatal rat cardiomyocytes

Mated female Sprague Dawley rats (>12 weeks old) were obtained from Janvier Labs and housed under standard conditions with controlled dark–light cycle, temperature and humidity in cages at the animal facility of the Goethe University Clinics Frankfurt. Rats were killed by cervical dislocation, and hearts were obtained from rat pups at P1 and P2 according to the current law of Hessen. Hearts were then transferred into Hank’s buffered saline solution (without Ca^2+^ or Mg^2+^) containing 0.2% 2,3-butanedione monoxime (short BDM, Sigma-Aldrich, B0753-25G) and cut into small pieces. Tissue dissociation was performed in 5 ml of a commercially available enzyme mix (Neonatal Heart Dissociation Kit, mouse and rat from Miltenyi Biotec, 130-098-373). To dissociate solid heart tissue, the gentleMACS Dissociator (Miltenyi Biotec) with the preprogrammed program ‘m_neoheart_01_01’ was used after each of the four digestion steps for 15 min at 37 °C. Cardiomyocytes and fibroblasts in the digested heart suspension were pelleted by centrifugation (80*g*, 5 min), resuspended in plating medium (DMEM high glucose, M199 EBS (both without l-glutamine, BioConcept), 10% horse serum (Thermo Fisher Scientific), 5% FCS (Thermo Fisher Scientific), 2% l-glutamine (Thermo Fisher Scientific) and penicillin–streptomycin (Thermo Fisher Scientific)), plated in 6-cm cell culture dishes (Greiner Bio-One) and incubated for 100 min at 37 °C with 5% CO_2_ in a humidified atmosphere. As fibroblasts attach to uncoated culture dishes, cardiomyocytes were taken from the culture supernatant.

Cardiomyocytes were cultured in maintenance medium (DMEM high glucose, M199 EBS (both without l-glutamine by BioConcept), 1% horse serum, 2% l-glutamine and penicillin–streptomycin) at 37 °C with 5% CO_2_ in a humidified atmosphere. Before plating, cell culture dishes were coated with 0.3 mg ml^−1^ collagen (354236, Corning) for 1 h at room temperature. One day after isolation, the medium of cultured cardiomyocytes was changed to maintenance medium containing no treatment, 200 µM PE (P6126-5G, Merck), 10 µg ml^−1^ recombinant EFNB2–Fc protein (rec-EFNB2, 7397-EB-050, R&D Systems) or PE with the recombinant EFNB2 protein for 72 h. For mRNA analysis, cells were collected in QIAzol Lysis Reagent (79306, Qiagen). For hypertrophic cell size assessment, cardiomyocytes were fixed with 4% paraformaldehyde (in DPBS, 28906, Thermo Fisher Scientific).

### Endothelial cell culture

HUVECs were purchased from PromoCell and cultured in endothelial cell basal medium (EBM, CC-3121, Lonza) supplemented with EGM-SingleQuots (CC-4133, Lonza) and 10% FBS (FCS, 10270-106, Gibco) at 37 °C with 5% CO_2_. HUVECs were routinely checked for mycoplasma.

### Cardiomyocyte–endothelial co-culture model

In total, 15,300 HUVECs were seeded onto eight-chamber glass slides (80826, Ibidi) coated with 1 µg ml^−1^ human fibronectin (F0895, Sigma-Aldrich), or 37,000 HUVECs were seeded in a 24-well plate (662-160, Greiner) and were transfected 24 h later with siRNA species using the RNAiMAX transfection reagent in Opti-MEM (S1985-026, Gibco) according to the manufacturer’s protocol (0.33 µl Lipofectamine RNAiMAX, 5653L, Invitrogen). siRNA pools (ON-TARGETplus Non-targeting Control Pool, D-001810-10-05; or ON-TARGETplus Human EFNB2 Smart Pool, L-003659-00-0005; Horizon) were used at a final concentration of 40 nM. The transfection mix was replaced with EBM medium containing all supplements and 10% FCS after 4 h.

After transfection (24 h), 6,000 (eight-chamber slides) or 100,000 (24-well plates) primary isolated neonatal rat cardiomyocytes were seeded on top of the endothelial layer. Cells were cultured in EBM with all supplements containing 10% FCS and plating medium at a ratio of 1:1. The plating medium was replaced with maintenance medium 24 h after seeding, supplemented with or without either recombinant EFNB2–Fc protein (10 µg ml^−1^) or PE (200 µM) for 72 h.

The cardiomyocyte contraction rate was determined 72 h after cardiomyocyte plating by using a bright-field microscope and counting the number of bpm. Cells were fixed with 4% paraformaldehyde (in DPBS, 28906, Thermo Fisher Scientific) for immunofluorescence analysis or lysed with the QIAzol Lysis Reagent (79306, Qiagen) for mRNA expression analysis.

### EPHB1 overexpression

Murine *Ephb1* (ORF transcript variant 1 or isoform 1) was amplified by PCR from a heart–brain cDNA pool and cloned into an ssAAV genome plasmid under transcriptional control of the human cardiac troponin T promoter (*TNNT2*)^46^. AAV6 vectors were generated by cotransfecting the genome plasmid and the adenoviral helper plasmid pDP6rs into low-passage HEK293T cells (ATCC, CRL-11268)^47^. This cell line has been authenticated by the company. AAV6 vectors were collected from cell culture supernatant and cell lysates, purified by discontinuous iodixanol gradient ultracentrifugation and titrated via quantitative real-time PCR^46,47^. In total, 50,000 HUVECs were seeded on eight-chamber glass slides that were coated with 1 µg ml^−1^ human fibronectin. After 5 h, 6,000 primary isolated neonatal rat cardiomyocytes were seeded on top of the endothelial layer and cultured in EBM with all supplements containing 10% FCS and plating medium at a ratio of 1:1. After seeding cardiomyocytes (24 h), cells were transduced with AAV6 vector particles delivering either *EGFP* (control) or *Ephb1* (1 × 10^5^ vg per cell), replacing the plating medium with maintenance medium. After AAV6-mediated transduction (24 h), cells were treated with or without 200 µM PE. The PE stimulation was repeated after 48 h for an additional 24 h before preparing cells for immunofluorescence staining.

### Immunofluorescence for cell culture

For hypertrophy assessment, cells were fixed with 4% paraformaldehyde, treated with 0.2% Triton X-100 (Merck) in DPBS for 10 min and blocked with 2% donkey serum (ab7475, Abcam), 3% BSA (8076.2, Carl Roth) and 0.2% Triton for 1 h. Cells were stained with anti-α-actinin (1:200, A7811, Merck) and anti-CDH5 (1:200, 2500S, Cell Signaling) antibodies in DPBS with 2% donkey serum, 3% BSA and 0.2% Triton overnight at 4 °C. After three washing steps with DPBS, samples were incubated with phalloidin (1:100, O7466, Thermo Fisher), DAPI (1:1,000, D9542, Merck) and secondary antibodies (1:200 anti-ms-647, A32728, Thermo Fisher; 1:200 anti-rb-555, A32794, Thermo Fisher) for 1 h at room temperature. Finally, cells were washed three times with DPBS and kept in DPBS at 4 °C until imaging. Imaging was performed using the Leica TCS SP8 confocal microscope with LAS X software (version 2.0.2), and Volocity software (version 6) was used for quantification by analyzing the size of 20–40 cardiomyocytes per condition. Cardiomyocyte cell area was detected by the immunofluorescence signal for α-actinin.

### Western blot experiments

Human cardiomyocyte ventricular primary cells (36044-15VT, Celprogen) were cultured at 37 °C with 5% CO_2_ in human cardiomyocyte serum-free medium (M36004-15, Celprogen) supplemented with 5% FCS. In total, 250,000 cells were seeded into a six-well plate (657160, Greiner) coated with 1 µg ml^−1^ human fibronectin. The medium was changed after 24 h. After an additional 24 h, cells were starved with cardiomyocyte medium without serum for 1 h and subsequently stimulated with or without 10 µg ml^−1^ recombinant EFNB2–Fc protein for 15 min. Cells were washed with ice-cold DPBS, snap frozen in liquid nitrogen and lysed in RIPA buffer (R0278, Sigma-Aldrich) supplemented with protease-inhibitor cocktail (11852700, Roche) and phosphatase inhibitor (04906837001, Roche). After a 45-min incubation on ice, lysates were centrifuged at 14,000*g* for 15 min at 4 °C. Protein concentrations were determined by the Bradford assay using the ROTIQuant assay (K015.1, Carl Roth). Protein (30 µg) reduced with Laemmli SDS (6×, J61337, Alfa Aesar) was separated on a Mini-PROTEAN TGX gel (4561094, Bio-Rad) and transferred onto a nitrocellulose membrane using a semi-dry fast blot (high MW, 25 V, 1.3 A, 10 min, Power Blotter, Invitrogen) according to the manufacturer’s protocol. Membranes were blocked for 1 h in blocking solution (5% Blotto nonfat milk (sc-2325, Santa Cruz) in Tris-buffered saline supplemented with 0.05% Tween-20). Primary antibodies were incubated overnight at 4 °C in blocking solution (1:1,000 anti-phosphorylated EPHB1, PA5106132, Invitrogen; 1:1,000 anti-phosphorylated EPHA4, PA5105119, Invitrogen; 1:5,000 anti-α-tubulin-1B chain, ab6160, Abcam). Secondary antibodies were incubated for 1 h at room temperature (1:1,000 donkey anti-rat HRP, ab102182, Abcam; 1:1,000 donkey anti-rabbit HRP, ab6802, Abcam). Proteins were detected based on HRP substrate-based enhanced chemiluminescence (WBKLS0500, Millipore), visualized using FusionCapt Advance Solo 4 software (version 16.15) and quantified using ImageJ (version 1.52g).

### Patient material for histological validation

Frozen biopsies from four non-hypertrophied hearts and five hypertrophied hearts (characteristics are summarized in Supplementary Table 3) were embedded in OCT and stored at −80 °C until sectioning. Hypertrophied myocardial tissues were obtained from five patients with hypertrophied cardiomyopathy. The diagnosis of hypertrophied cardiomyopathy was confirmed with a left ventricular wall thickness measurement >15 mm in one or more left ventricular segments after severe valvular heart disease was excluded by cardiac imaging (echocardiography and/or magnetic resonance imaging). Biopsy specimens were obtained from the apical part of the free left ventricular wall from patients undergoing cardiac catheterization using a standardized protocol. Biopsies 1–2 mm in diameter were immediately washed in ice-cold saline (0.9% NaCl), and leftover biopsies were transferred to and stored in liquid nitrogen.

All participants gave written informed consent (application number S-390/2011). The study was approved by an institutional review committee of the University Heidelberg in compliance with internal standards of the German government, and procedures followed were in accordance with institutional guidelines and the Declaration of Helsinki.

### Transverse aortic constriction model

For further experimental validation of bioinformatic findings, a mouse model of TAC-induced hypertrophy was employed. C57BL/6J male (12-week-old) mice were obtained from Janvier Labs and housed under standard conditions in cages at the animal facility of the Max Planck Institute for Heart and Lung Research in Bad Nauheim, Germany with controlled dark–light cycle, temperature and humidity.

After 2 weeks of adaptation, mice were randomly divided into two groups and underwent sham or TAC surgery. For TAC surgery, *n* = 6 mice were anesthetized with 5% isoflurane gas (in 100% oxygen) in an induction chamber, followed by intubation. Anesthesia was maintained throughout the surgery at 1.5% isoflurane in 100% oxygen while keeping the body temperature at 37 °C ± 0.5 °C using a heat pad monitored by a rectal thermometer probe. Before anesthesia, 0.1 mg per kg body weight of buprenorphin was injected subcutaneously. The surgical level of anesthesia was confirmed by the absence of a toe pinch reflex. The skin of the upper thorax was prepared for surgery by shaving and disinfection with povidone iodine–alcohol, after which 1 mg per kg bupivacain was injected into the three cranial intercostal spaces. Thoracotomy was performed at the left side by cutting the first to third ribs close to the sternum. Ribs were retracted using a fine retractor to expose the aorta. A 6-0 silk suture was placed around the transverse aorta between the left and right carotic artery and tied loosely into a single knot. A presterilized, blunt-ended 26G needle was placed within the silk knot, which was then tightened fully, encircling the transverse aorta and the blunt-ended needle. The suture was secured with a double knot before the blunt-ended needle was removed. The incision was closed in layers using 5-0 sutures. The sham operation only included thoracotomy without applying constriction (*n* = 4). Hearts were collected 5 weeks after surgery, perfused with saline, frozen in liquid nitrogen and stored at −80 °C. For analysis, hearts were embedded in OCT.

All procedures were performed in accordance with German animal-protection laws and EU (directive 2010/63/EU) ethical guidelines and were approved by the local governmental animal-protection authority Regierungspräsidium Darmstadt (TVA B2/1208).

### Human cardiac organoid formation

Human induced pluripotent stem cells (iPSCs) (WTSli081-A cell line, 66540196, EBiSC) were used to generate cardiac organoids. In brief, 500 human iPSCs were cultured on an ultra-low-attachment surface in TeSR-E8 medium (05990, Stemcell Technologies) at 37 °C with 5% CO_2_ in a humidified atmosphere to form iPSC aggregates. After 2 d, iPSC aggregates were differentiated into cardiac organoids (hCOs) using the STEMdiff Cardiomyocyte Differentiation kit (05010, Stemcell Technologies), following instructions from the supplier. hCOs were then maintained in mixed medium consisting of STEMdiff Cardiomyocyte Maintenance Basal Medium (05020, Stemcell Technologies) and Endothelial Cell Growth Medium 2 (C-22111, PromoCell) at a ratio of 4:1, refreshing the medium every 2nd day for a further 28 d. After hCOs were ready to use, the medium was changed to medium supplemented with or without 200 µM PE, 10 µg ml^−1^ recombinant EFNB2–Fc protein or PE with recombinant EFNB2 for 72 h. For further immunohistological analysis, hCOs were fixed with Histofix for 1 h at room temperature, embedded in OCT and stored at −80 °C until sectioning.

### Fluorescence immunohistochemistry

For immunofluorescence staining of patient biopsies, mouse hearts and human cardiac organoids, 5-µm cryosections (10 µm for organoids) were cut at the cryostat (Leica, CM1950). Cryosections were fixed with Histofix for 10 min at room temperature (no fixation for organoid sections) and subsequently washed using DPBS containing 0.2% Triton X-100 (hereafter referred to as DPBST) for 5 min at room temperature. Sections were blocked with 5% donkey serum in DPBST for 1 h at room temperature. Primary antibodies were diluted in blocking solution and incubated overnight at 4 °C (1:50 anti-EPHB1, PA5-111626, Thermo Fisher Scientific; 1:50 anti-VEGFB, PA5-116113, Thermo Fisher Scientific; 1:200 anti-α-actinin, A7811, Merck). Next, sections were washed three times (see above). Secondary antibodies were diluted in blocking solution and incubated for 1 h at room temperature in the dark (1:200 anti-rabbit 555 antibody, A-31572, Thermo Fisher Scientific; 1:300 wheat germ agglutinin, W32466, Thermo Fisher Scientific). After washing (see above), cryosections were mounted with ProLong Gold Antifade Mountant (Thermo Fisher Scientific) supplemented with DAPI (1:1,000, Carl Roth). Imaging was performed with the Leica TCS SP8 confocal microscope, and Volocity software (version 6) was used for quantification. Cardiomyocyte size in cardiac organoids was determined based on a combination of the α-actinin signal, identifying 50 cardiomyocytes per organoid, and the wheat germ agglutinin signal, marking cell borders, using ImageJ software (version 1.52g).

### Ribonucleic acid analysis

Cells were lysed with QIAzol Lysis Reagent (79306, Qiagen), and RNA was isolated using the miRNeasy Micro kit (1071023, Qiagen) according to the manufacturer’s protocol (2021) with additional DNase I digestion (79254, Qiagen) for 15 min. The quality and concentration of the isolated RNA was assessed and measured with the NanoDrop 2000 spectrophotometer from Thermo Fisher Scientific. mRNA (250–1,000 ng) was reverse transcribed using random hexamer primers (N808-0261, Life Technologies) and M-MLV Reverse Transcriptase (N8080018, Life Technologies).

cDNA was analyzed using the real-time quantitative PCR method. cDNA was amplified with the Fast SYBR Green Mastermix (4385612, Life Technologies) and specific primers (Vegfa (forward, ACCATGCCAAGTGGTGAAGT; reverse, GACCCAAAGTGCTCCTCGAA), Vegfb (forward, TTTCCACGGGCTTTACACGA; reverse, GTGCTGTCATGCCTTGTTCC), Ephb1 (forward, CCCACAGCATGTCTCTGTCA; reverse, AAGGTGATGCTCCTCATGGT), Nppa (forward, CCCGTATACAGTGCGGTGTC; reverse, TCAATCCTACCCCCGAAGCA), Nppb (forward, GGCTCAGAGACAGCTCTCAA; reverse, CCGATCCGGTCTATCTTCTGC), Acta1 (forward, GCACCGCAAATGCTTCTAGG; reverse, CGATGGTCGATTGTCGTCCT)) on a ViiA 7 Real-time qPCR System (Life Technologies). Expression levels were normalized to the housekeeping gene *Hprt1* (forward, CCTCCTCCGCCAGCTT; reverse, GTCATAACCTGGTTCATCATCACT) or rat-specific *Hprt1* (forward, GTCCCAGCGTCGTGATTAGT; reverse, CTTGCCGCTGTCTTTTAGGC) according to the $2^{-{\Delta}_{c_t}}$method using QuantStudio Real-Time PCR software (version 1.3).

### Statistical analysis

Data are represented as mean and error bars, indicating s.e.m. Data were assessed statistically for normality (Shapiro–Wilk and Kolmogorov–Smirnov tests). Statistical significance for data with a Gaussian distribution was determined using two-sided, unpaired Student’s *t*-tests for two-group comparison. For data not following a Gaussian distribution, statistical analysis was performed using Wilcoxon rank-sum tests for two-group comparison. For comparison of more than two groups, one-way ANOVA with a post hoc Tukey test was used for data with a Gaussian distribution (Dunnett’s correction for pairwise comparisons), and the Kruskal–Wallis test with a post hoc Dunn’s test was used for data not following a Gaussian distribution. To detect significant outliers, Grubb’s *t*-test was used (*P* < 0.05). For snRNA-seq analysis, adjusted *P* values were calculated with the Seurat function ‘FindAllMarkers’. For a more robust *P*-value calculation, we applied the cluster *t*-test from the package ‘Hmisc’. Here, we defined the condition (healthy versus AS) as the group parameter and sample IDs as the cluster parameter in the ‘t.test.cluster()’ function. Linear regression analysis of snRNA-seq data was performed using the stats (version 3) function ‘lm()’, determining the coefficient of determination (*R*^2^). *A P* value <0.05 was considered statistically significant. Statistical analysis was performed using R (version 4.0.3) or Prism (version 9.2.0). Figure panels were created using Inkscape (version 0.92).

### Reporting Summary

Further information on research design is available in the Nature Research Reporting Summary linked to this article.

### Supplementary information

Supplementary Information Uncropped Western Blot images for Fig 3f and ED Fig8f; Image files 3i,3j, 4d,4e,6d,9a

Reporting Summary

Supplementary Tables Supplementary Tables 1–3.

### Source data

Source Data Fig. 3 Raw data quantification.

Source Data Fig. 3 Raw images for Fig. 3i,j.

Source Data Fig. 4 Raw data quantification for Fig. 4.

Source Data Fig. 4 Raw images for Fig. 4c–e.

Source Data Extended Data Fig. 5 Raw data quantification for Extended Data Fig. 5a.

Source Data Extended Data Fig. 6 Raw data quantification for Extended Data Fig. 6a–c.

Source Data Extended Data Fig. 6 Raw images for Extended Data Fig. 6d.

Source Data Extended Data Fig. 8 Raw data quantification for Extended Data Fig. 8f.

Source Data Extended Data Fig. 8 Raw image for Extended Data Fig. 8f.

Source Data Extended Data Fig. 9 Raw data quantification for Extended Data Fig. 9a–c.

Source Data Extended Data Fig. 9 Raw images for Extended Data Fig. 9a.

## Data Availability

The datasets generated during and/or analyzed during the current study are available at the ArrayExpress data repository with the accession number E-MTAB-11268. The used snRNA-seq dataset of the septum from healthy heart samples was taken from the Litvinukova et al. study^10^ (https://www.heartcellatlas.org/#DataSources). Source data are provided with this paper.

## Code availability

The full code used during the current study is available at https://github.com/djhn75/DiseaseHeartCellAtlas.
