# Supplementary material for: A human cell atlas of the pressure-induced hypertrophic heart
Source: Nat Cardiovasc Res. 2022 Feb 14;1(2):174–85. doi: 10.1038/s44161-022-00019-7 (PMC11357985; doi:10.1038/s44161-022-00019-7)
Supplement: Supplementary file 1 — Uncropped Western Blot images for Fig 3f and ED Fig8f; Image files 3i,3j, 4d,4e,6d,9a [file 44161_2022_19_MOESM1_ESM.pdf]

nature portfolio

https://doi.org/10.1038/s44161-022-00019-7

Supplementary information

A human cell atlas of the pressure-induced hypertrophic heart

In the format provided by the authors and unedited

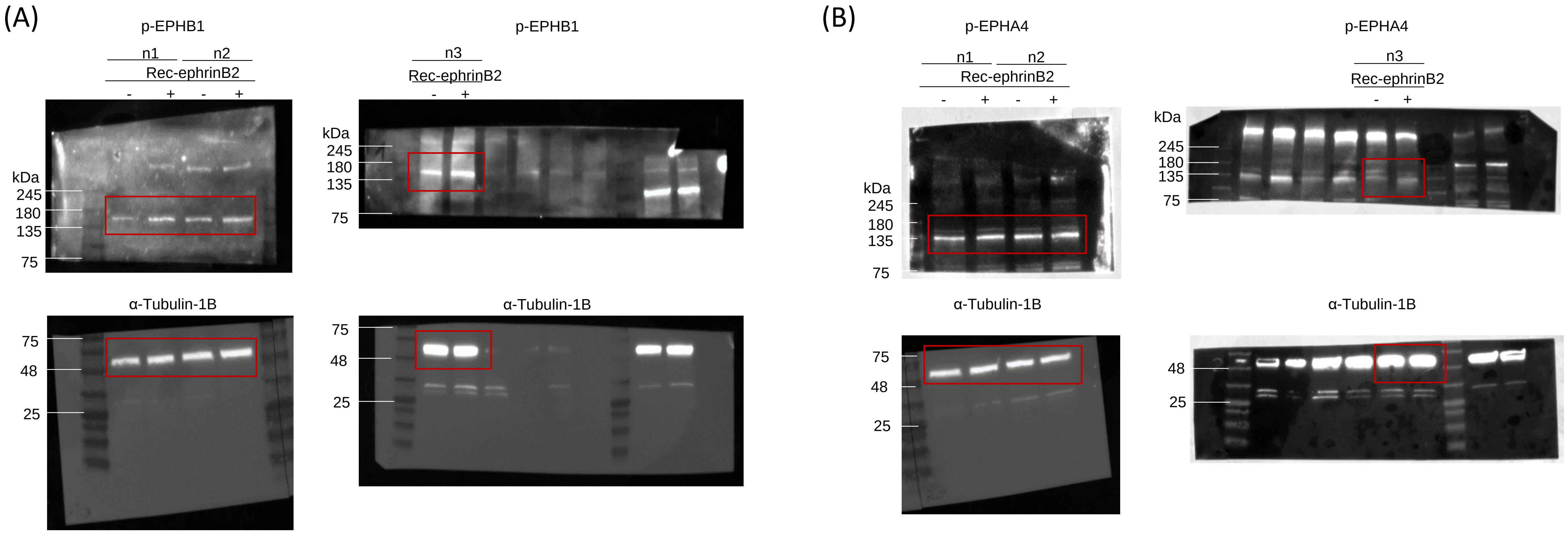

Supplementary Figure 1: **Raw uncropped images from Figure 4c (A) and Extended Data Figure 8f (B).**

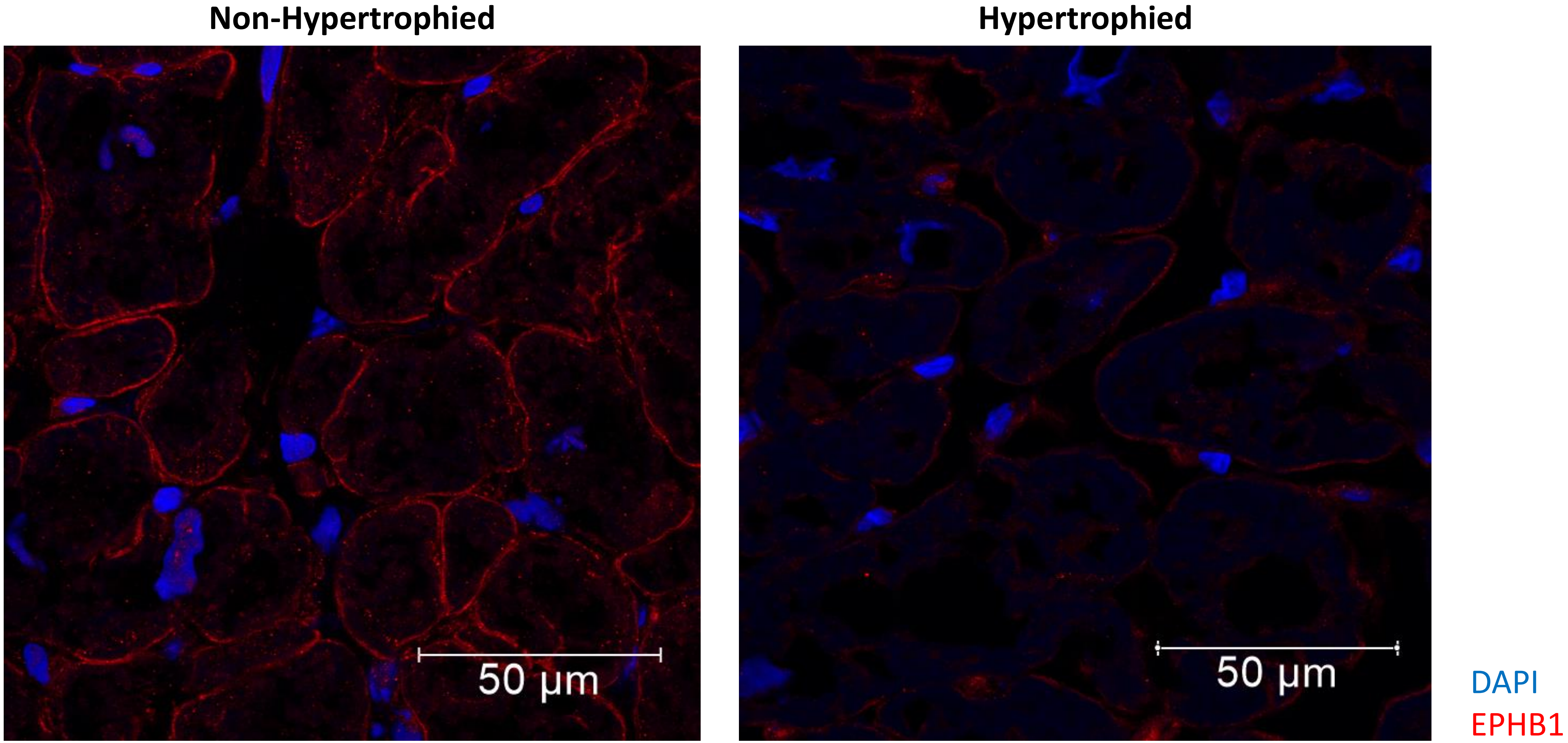

Supplementary Figure 2: **Raw uncropped images from Figure 3i.**

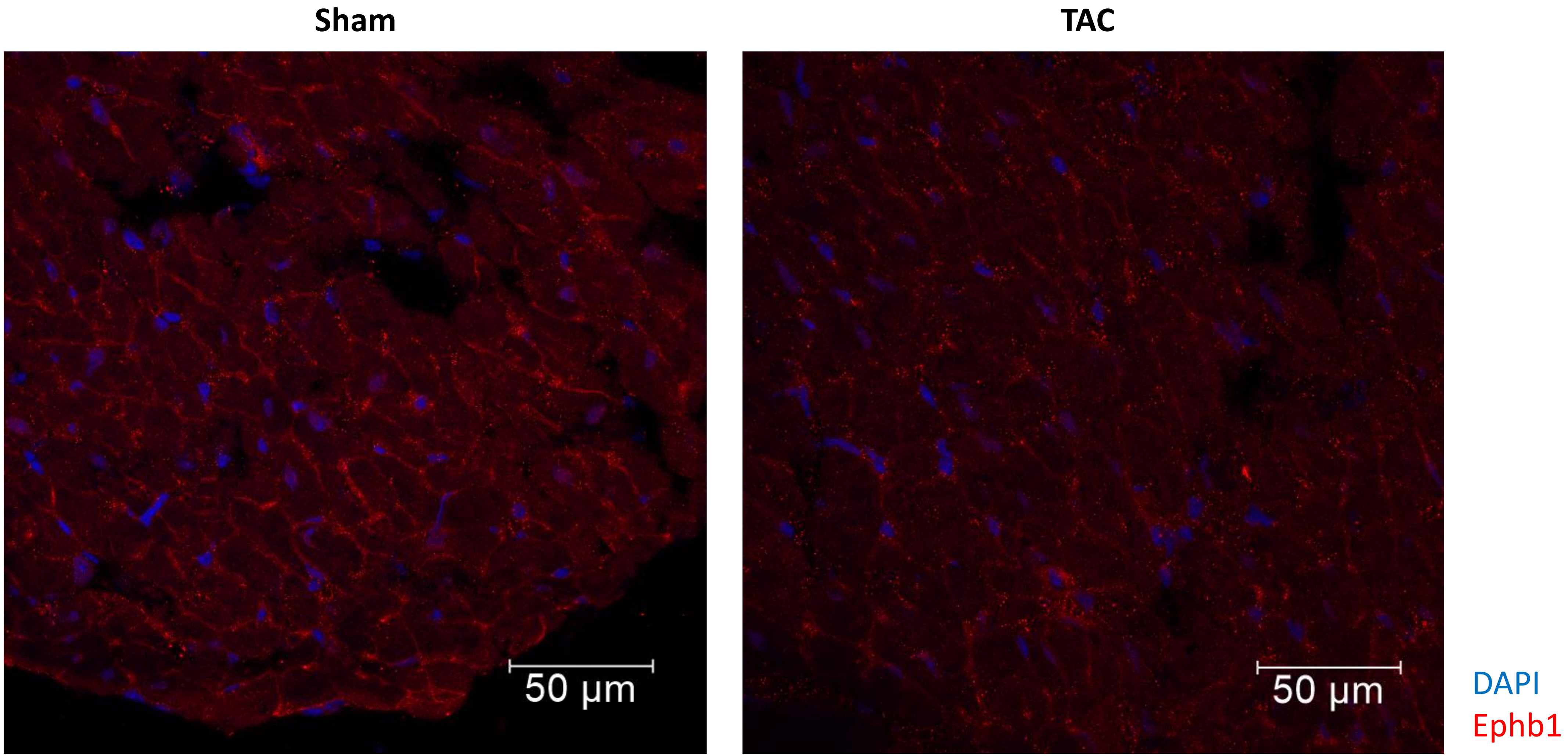

Supplementary Figure 3: **Raw uncropped images from Figure 3j.**

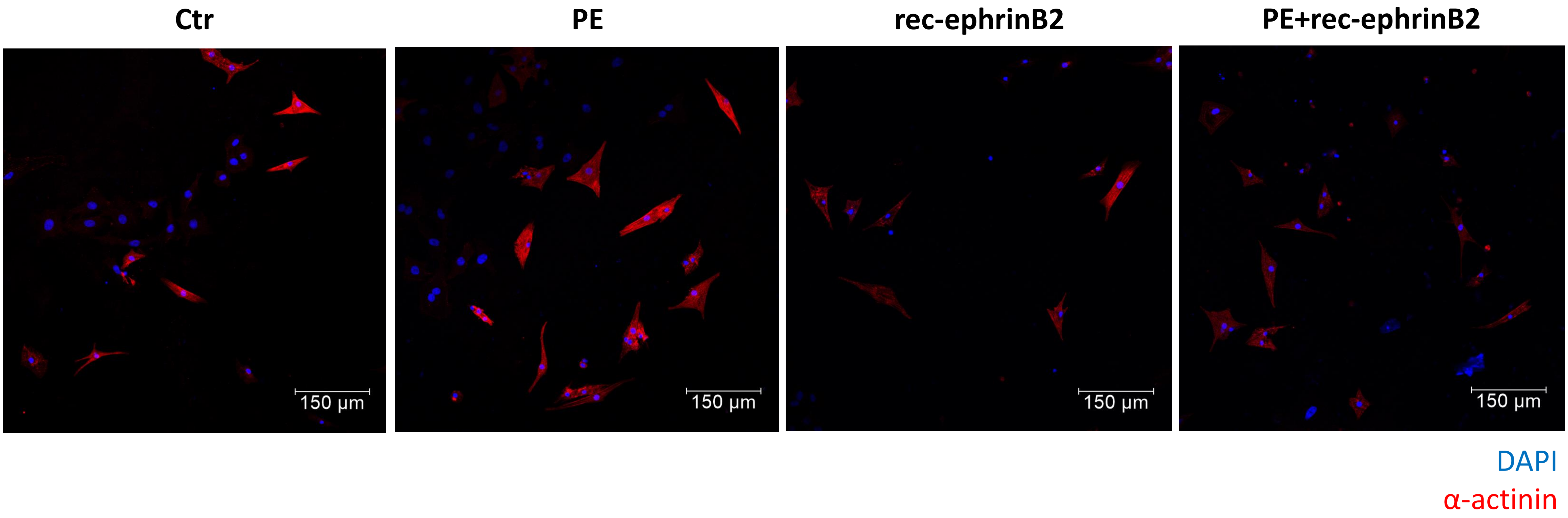

Supplementary Figure 4: **Raw uncropped images from Figure 4d.**

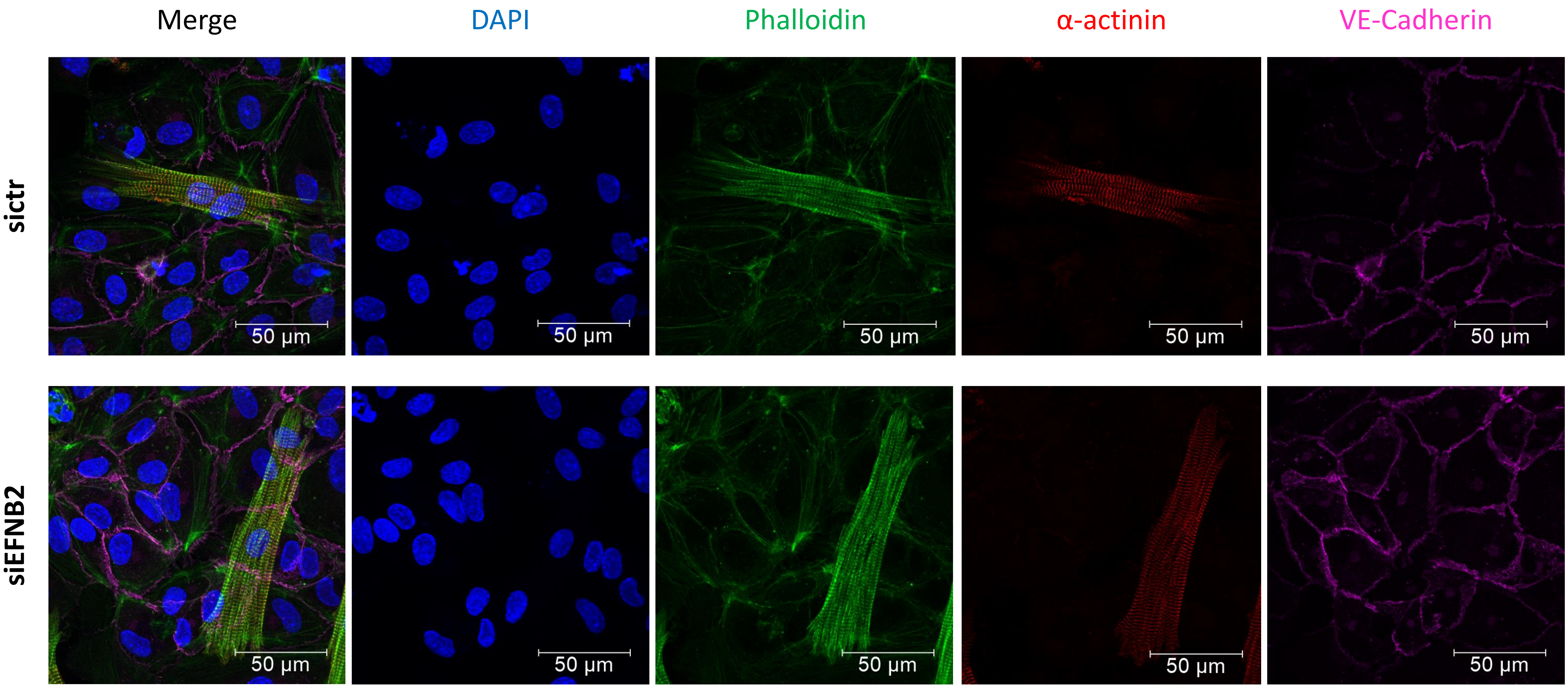

Supplementary Figure 5: **Raw uncropped images from Figure 4e.**

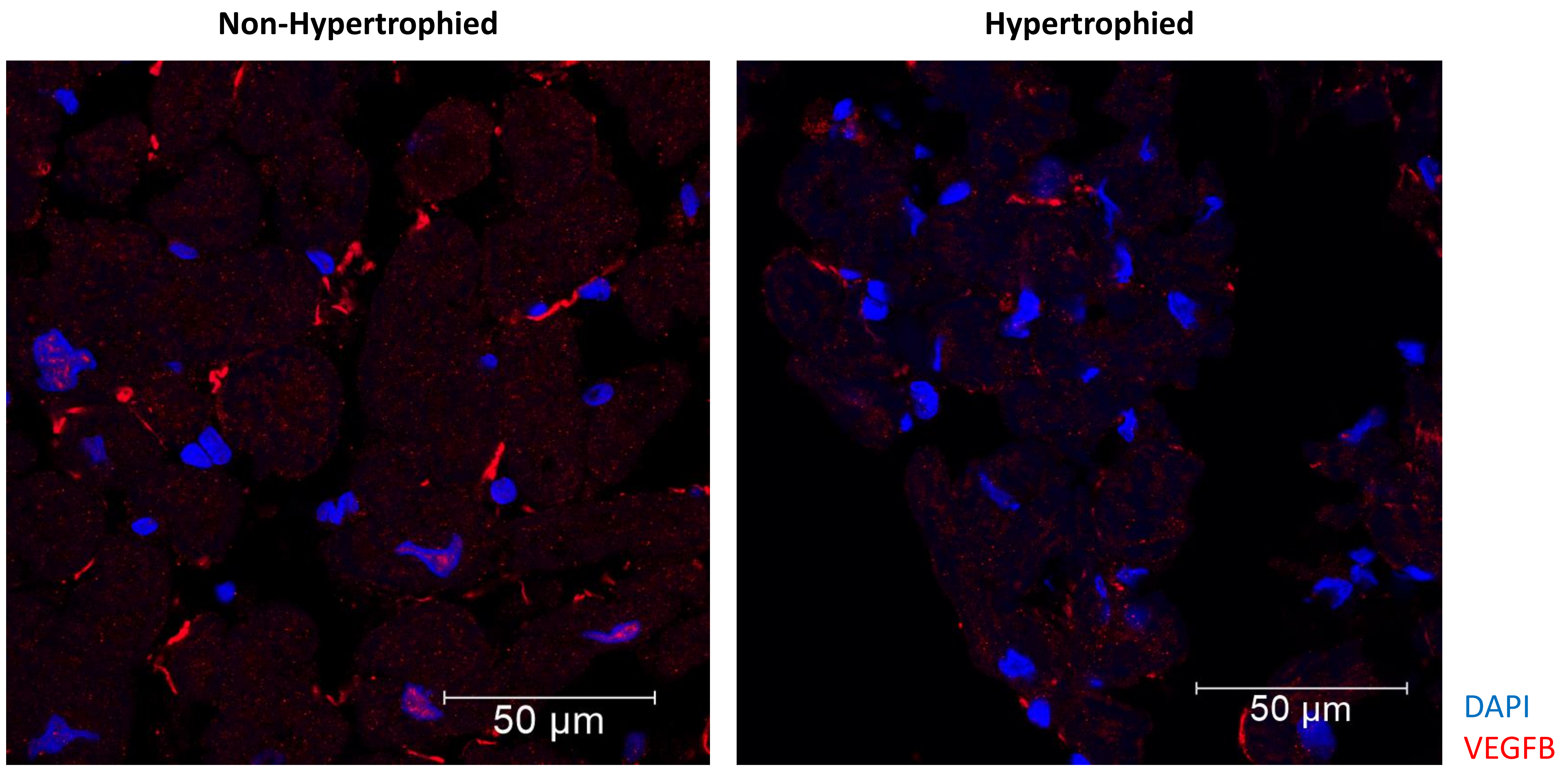

Supplementary Figure 6: **Raw uncropped images from Extended Data Figure 6d.**

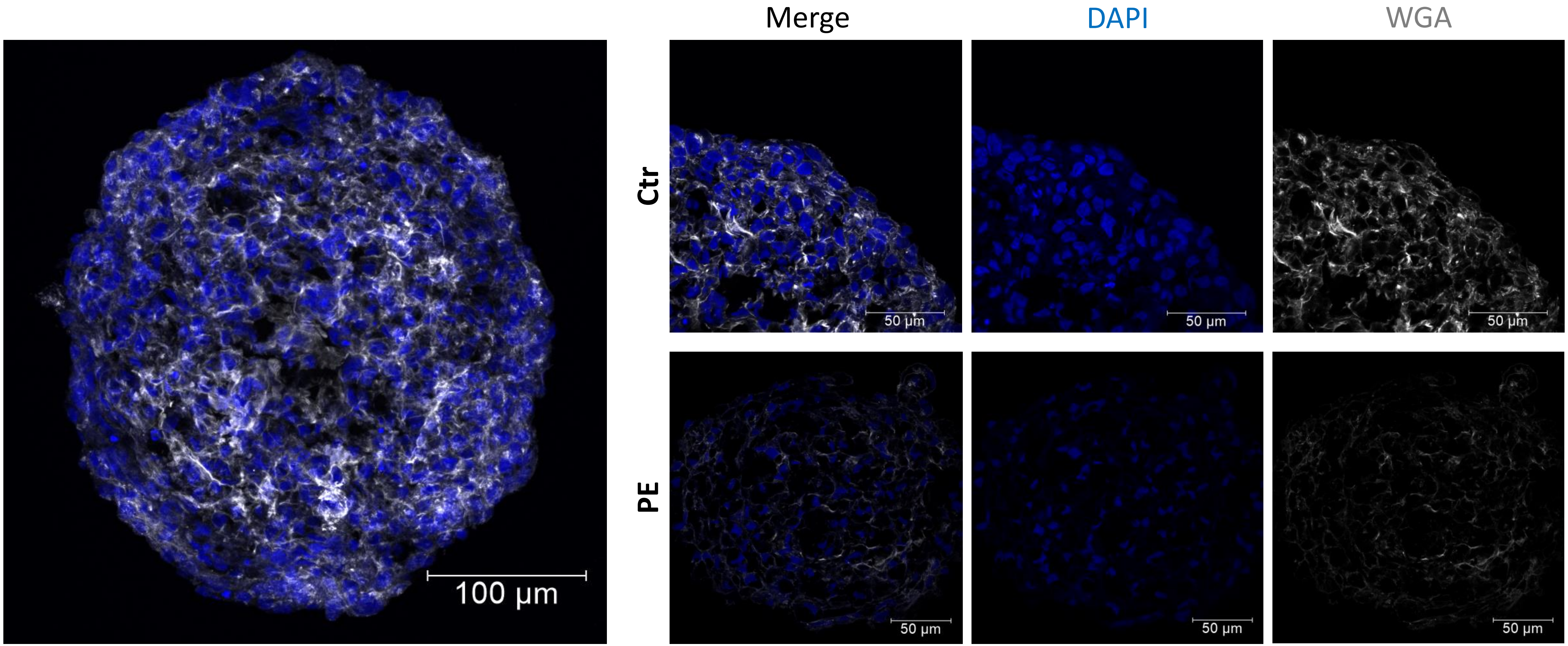

Supplementary Figure 7: **Raw uncropped images from Extended Data Figure 9a.**
