# Supplementary material for: A human cell atlas of the pressure-induced hypertrophic heart
Source: Nat Cardiovasc Res. 2022 Feb 14;1(2):174–85. doi: 10.1038/s44161-022-00019-7 (PMC11357985; doi:10.1038/s44161-022-00019-7)
Supplement: Supplementary file 2 — Reporting Summary [file 44161_2022_19_MOESM2_ESM.pdf]

nature portfolio

Corresponding author(s): Prof. Dr. Stefanie Dimmeler

Last updated by author(s): Dec 30, 2021

Reporting Summary

Nature Portfolio wishes to improve the reproducibility of the work that we publish. This form provides structure for consistency and transparency in reporting. For further information on Nature Portfolio policies, see our Editorial Policies and the Editorial Policy Checklist.

Statistics

For all statistical analyses, confirm that the following items are present in the figure legend, table legend, main text, or Methods section.

n/a	Confirmed	
☐	☒	The exact sample size (*n*) for each experimental group/condition, given as a discrete number and unit of measurement
☐	☒	A statement on whether measurements were taken from distinct samples or whether the same sample was measured repeatedly
☐	☒	The statistical test(s) used AND whether they are one- or two-sided<br>*Only common tests should be described solely by name; describe more complex techniques in the Methods section.*
☒	☐	A description of all covariates tested
☐	☒	A description of any assumptions or corrections, such as tests of normality and adjustment for multiple comparisons
☐	☒	A full description of the statistical parameters including central tendency (e.g. means) or other basic estimates (e.g. regression coefficient) AND variation (e.g. standard deviation) or associated estimates of uncertainty (e.g. confidence intervals)
☐	☒	For null hypothesis testing, the test statistic (e.g. *F*, *t*, *r*) with confidence intervals, effect sizes, degrees of freedom and *P* value noted<br>*Give P values as exact values whenever suitable.*
☒	☐	For Bayesian analysis, information on the choice of priors and Markov chain Monte Carlo settings
☒	☐	For hierarchical and complex designs, identification of the appropriate level for tests and full reporting of outcomes
☐	☒	Estimates of effect sizes (e.g. Cohen's *d*, Pearson's *r*), indicating how they were calculated

Our web collection on statistics for biologists contains articles on many of the points above.

Software and code

Policy information about availability of computer code

Data collection	Immunofluorescence images were aquired using the LAS X software (version 2.0.2). We processed single cell data with the CellRanger (10X Genomics) suite (version 3.0.1). Western Blot images were aquired using the FusionCapt Advance Solo4 software (version 16.15). RNA expression data was collected using the QuantStudio Real-Time PCR software (version 1.3).
Data analysis	For secondary single cell analsysis we used R software (version 4.0.3) and packages written in R for data analysis and visualization: ggplot2 (version 3.3.0), RColorBrewer (version 1.1-2), pheatmap (version 1.0.12), dplyr (version 0.8.5), reshape2 (version 1.4.4), tidyr (version 1.0.2), Rcpp (version 1.0.4.6), scales (version 1.1.0), ggpubr (version 0.1.9), Matrix (version 1.2.-18), Seurat (version 4.0.2), viridis (version 0.5.1), DoubletFinder (version 2.0.3), LISI (version 1.0), rstatix (version 0.6.0), Hmisc (version 4.4.0), CellphoneDB (version 2.0), CellChat (version 1.1.43), stats (version 4.0.3).<br><br>Immunofluorescence and Western Blot images were analysed using Volocity (version 6) and ImageJ (version 1.52g).<br><br>RNA expression data was analyzed using the QuantStudio Real-Time PCR software (version 1.3).<br><br>GoTerm Analysis were analyzed using Metascape (2019; PMID: 30944313)<br><br>Other data was analysed and visualized using R (version 4.0.3) or Prism (version 9.2.0). Figure panels were created using Inkscape (version 0.92).

For manuscripts utilizing custom algorithms or software that are central to the research but not yet described in published literature, software must be made available to editors and reviewers. We strongly encourage code deposition in a community repository (e.g. GitHub). See the Nature Portfolio guidelines for submitting code & software for further information.

Data

Policy information about availability of data

All manuscripts must include a data availability statement. This statement should provide the following information, where applicable:

- Accession codes, unique identifiers, or web links for publicly available datasets
- A description of any restrictions on data availability
- For clinical datasets or third party data, please ensure that the statement adheres to our policy

The datasets generated during and/or analyzed during the current study are available at the array express data depository with the accession number E-MTAB-11268.The used snRNA sequencing dataset of the septum from healthy heart samples were taken from Litvinukova et al (https://www.heartcellatlas.org/#DataSources).

Field-specific reporting

Please select the one below that is the best fit for your research. If you are not sure, read the appropriate sections before making your selection.

☒ Life sciences ☐ Behavioural & social sciences ☐ Ecological, evolutionary & environmental sciences

For a reference copy of the document with all sections, see nature.com/documents/nr-reporting-summary-flat.pdf

Life sciences study design

All studies must disclose on these points even when the disclosure is negative.

Sample size

The samples size of human biopsies used for the single-nuclei RNA-sequencing analysis and its validation is based on the availability of the tissue. However, we report high sample sizes above the average of the field (nuclei & number of patients)
Recent examples of human heart tissue:
10.1038/s41556-019-0446-7 N=21422
10.1038/s41467-018-06639-7 N=1190
10.1161/CIRCULATIONAHA.120.051391 N=18211

The murine heart samples were available from left-over histology samples of previously published studies (PMID: 31399804; n=4-10).
For human heart samples we used all samples that were available to us from the biobank of our collaborators.
For in vitro experiments we estimated sample size based on previous experiments performed by us or others, which showed significant increase of hypertrophy and stress markers in cardiomyocyte cultures upon stimulation.
e.g.
Badorff et al Dimmeler, J Clin Invest 2002 Feb;109(3):373-81. (n=3)
Other studies:
Stress markers: https://doi.org/10.1093/cvr/cvaa233 (N=3)
Jentzsch et al, J Mol Cell Cardiol. 2012 Jan;52(1):13-20 (N=3-5)

Data exclusions

We excluded one batch of cardiomyocyte analysis in Figure 5d from our analysis based on a statistical outlier test (Grubb´s test $p < 0.05$). Furthermore, bad quality of this batch of freshly solated rat cardiomyoctes was strikingly by decreased cell size in all treatment groups compared to previous batches.

Replication

All replicates performed were included within the data shown in our manuscript and are described in the respective figure legend. Individual values are shown in each figure.

Experiments were generally performed in at least 3 biologically independent experiments to ensure reproducibility of the data. All samples were generated independently to achieve biological replicates, no technical replicates.

Only one batch of cardiomyocyte analysis in Figure 5d, which was of bad cell quality (strikingly decreased cell size in all treatment groups compared to previous batches) and was excluded based on a statistical outlier test (Grubb´s test $p < 0.05$).

We confirm, that all attemps at replication were successful.

Randomization

Mice have been randomly assigned to treatment cohorts. Cells have been randomly allocated to respective groups.

Blinding

All single-nuclei RNA-sequencing analysis were performed blinded, as they were analyzed in an unbiased fashion using unbiased algorithms as clustering or ligand-receptor analysis. Stainings of human and murine tissue sections were also blinded. Other experiments were non-blinded due to restricted number of researchers and poor feasibility.

Reporting for specific materials, systems and methods

We require information from authors about some types of materials, experimental systems and methods used in many studies. Here, indicate whether each material, system or method listed is relevant to your study. If you are not sure if a list item applies to your research, read the appropriate section before selecting a response.

Materials & experimental systems

n/a	Involved in the study
☐	☒ Antibodies
☐	☒ Eukaryotic cell lines
☒	☐ Palaeontology and archaeology
☐	☒ Animals and other organisms
☐	☒ Human research participants
☒	☐ Clinical data
☒	☐ Dual use research of concern

Methods

n/a	Involved in the study
☒	☐ ChIP-seq
☒	☐ Flow cytometry
☒	☐ MRI-based neuroimaging

Antibodies

Antibodies used

All antibodies are commercially purchased:

anti-sarcomeric-alpha-actinin (#A7811, monoclonal, Merck, Lot#0000120830; clone number EA-53; 1:200)
anti-VE-Cadherin (#2500S, monoclonal, Cell Signaling, Lot#6; clone number D87F2; 1:200)
anti-mouse 647 (#A32728, polyclonal, Thermo Fisher, Lot#WE322197; 1:200)
anti-rabbit 555 (#A32794, polyclonal, Thermo Fisher, Lot#UI284002; 1:200)
anti-phospho-EPHB1 (#PA5106132, polyclonal, Invitrogen, Lot#WI3388818;1:1000)
anti-phospho-EPHA4 (#PA5105119, polyclonal, Invitrogen, Lot#WI3388816A; 1:1000)
anti-Tubulin-alpha-1B-chain (#ab6160, monoclonal, abcam, Lot#GR3382008-1; clone number YL1/2; 1:5000)
anti-rat HRP (#ab102182, polyclonal, abcam, Lot#GR251066-11,1:1000)
anti-rabbit HRP (#ab6802, polyclonal, abcam, Lot#GR3249175-5;1:1000)
anti-EPHB1 (#PA5111626, polyclonal, Thermo Fisher, Lot#R98217; 1:50)
anti-VEGFB (#PA5116113, polyclonal, Thermo Fisher, Lot#WC32333009;1:50)
anti-rabbit 555 (#A31572, polyclonal, Thermo Fisher, Lot#2286312; 1:200)

Validation

Primary Antibodies were validated by the manufacturer and confirmed by specific labeling of target molecules or cell types:

anti-sarcomeric-alpha-actinin (https://www.sigmaaldrich.com/DE/de/product/sigma/a7811)
anti-VE-Cadherin (https://www.cellsignal.com/products/primary-antibodies/ve-cadherin-d87f2-xp-rabbit-mab/2500)
anti-phospho-EPHB1 (https://www.thermofisher.com/antibody/product/Phospho-EphB1-Tyr928-Antibody-Polyclonal/PA5-106132)
anti-phospho-EPHA4 (https://www.thermofisher.com/antibody/product/Phospho-EphA4-Tyr602-Antibody-Polyclonal/PA5-105119)
anti-Tubulin-alpha-1B-chain (https://www.abcam.com/tubulin-antibody-yl12-loading-control-ab6160.html)
anti-EPHB1 (https://www.thermofisher.com/antibody/product/EphB1-Antibody-Polyclonal/PA5-111626)
anti-VEGFB (https://www.thermofisher.com/antibody/product/VEGFB-Antibody-Polyclonal/PA5-116113)

Secondary Antibodies were validated by the manufacturer and have been tested in our experimental conditions to rule out unspecific signal:

anti-mouse 647 (https://www.thermofisher.com/antibody/product/Goat-anti-Mouse-IgG-H-L-Highly-Cross-Adsorbed-Secondary-Antibody-Polyclonal/A32728)
anti-rabbit 555 (https://www.thermofisher.com/antibody/product/Donkey-anti-Rabbit-IgG-H-L-Highly-Cross-Adsorbed-Secondary-Antibody-Polyclonal/A32794)
anti-rat HRP (https://www.abcam.com/donkey-rat-igg-hl-hrp-ab102182.html)
anti-rabbit HRP (https://www.abcam.com/donkey-rabbit-igg-hl-hrp-ab6802.html)
anti-rabbit 555 (https://www.thermofisher.com/antibody/product/Donkey-anti-Rabbit-IgG-H-L-Highly-Cross-Adsorbed-Secondary-Antibody-Polyclonal/A-31572)

Eukaryotic cell lines

Policy information about cell lines

Cell line source(s)

Human umbilical cord endothelial cells (HUVECs) were purchased from Promocell (#C-12203). Human cardiomyocyte ventricular primary cells were purchased from Celprogen (#36044-15VT). HEK293T cells (#CRL-11268) were purchased from ATCC. Human induced pluripotent stem cells (hiPSCs; WTSli081-A cell line; #66540196) were purchased from EBiSC.

Authentication

Not authenticated by the authors.

Mycoplasma contamination

HUVEC cells, human cardiomyocyte ventricular primary cells, HEK293T cells as well as hiPS cells were tested and negative for mycoplasma.

Commonly misidentified lines
(See ICLAC register)

HEK293T Cells (The quality control of the company was done by STR profiling. For our virus production experiments, this cell line was only used in a low passage of the original stock.)

Animals and other organisms

Policy information about studies involving animals; ARRIVE guidelines recommended for reporting animal research

Laboratory animals	For the isolation of neonatal rat cardiomyocytes, mated female Sprague Dawley (>12-weeks old) were obtained from Janvier Labs. Female and male pubs were used for isolation of cardiomyocytes<br>For TAC-stainings of murine cardiac sections, male 12-weeks old C57BL/67 were obtained from Janvier Labs.
Wild animals	This study did not involve wild animals.
Field-collected samples	This study did not involve field-collected samples.
Ethics oversight	All animal experiments were executed in agreement with the animal welfare guidelines, German national laws, and EU ethical guidelines (Directive 2010/63/EU). All animals experiments and study protocols were authorized by the competent authority (Regierungspräsidium Darmstadt, Hessen, Germany).

Note that full information on the approval of the study protocol must also be provided in the manuscript.

Human research participants

Policy information about studies involving human research participants

Population characteristics	All demographic and clinical characteristics of patients involved in this study are extensively described within the Supplemental Tables.
Recruitment	The recruitment was performed by experts included as authors within this study. Heart failure patients were recruited based on defined inclusion and exclusion characteristics:<br>For single cell sequencing study:<br>Inclusion criteria:<br>- aortic stenosis<br>- elective operation for diagnosis aortic stenosis<br>- age > 18 years<br>- consent to participate in the study<br>Exclusion criteria:<br>- lack of consent to participate<br>-pregnancy<br>- emergency operation<br>For validation by histology, we applied for the use of human samples by the "Probennutzungsantrag" for the use of healthy and patient derived heart tissue sections with no defined inclusion or exclusion criteria.
Ethics oversight	Single-Cell Sequencing Analysis: Informed consent was obtained from all patients. The study was approved by an institutional review committee of the University Hospital of the Johann Wolfgang Goethe University in compliance with internal standards of the German government and procedures followed were in accordance with institutional guidelines (Application 347/18) and the Declaration of Helsinki.<br><br>Validation on protein level: All participants have given written informed consent (application number S-390/2011). The study was approved by an insitutional review committee of the University Heidelberg in compliance with internal standards of the German government, and procedures followed were in accordance with institutional guidelines and the Declaration of Helsinki.

Note that full information on the approval of the study protocol must also be provided in the manuscript.
