# Supplementary material for: A human cell atlas of the pressure-induced hypertrophic heart
Source: Nat Cardiovasc Res. 2022 Feb 14;1(2):174–85. doi: 10.1038/s44161-022-00019-7 (PMC11357985; doi:10.1038/s44161-022-00019-7)
Supplement: Supplementary file 5 — Raw images for Fig. 3i,j. [file 44161_2022_19_MOESM5_ESM.pdf]

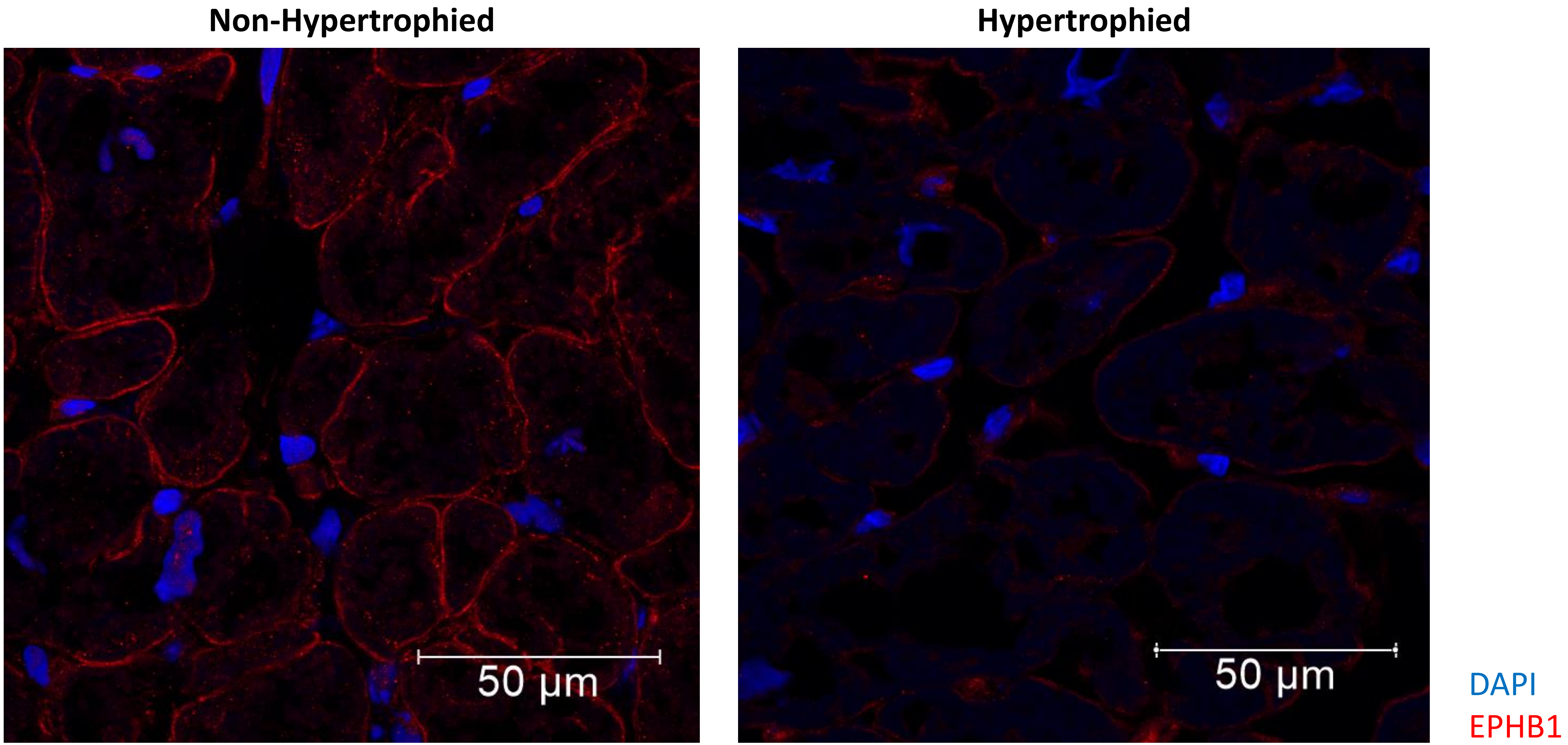

Raw uncropped images from Figure 3i.

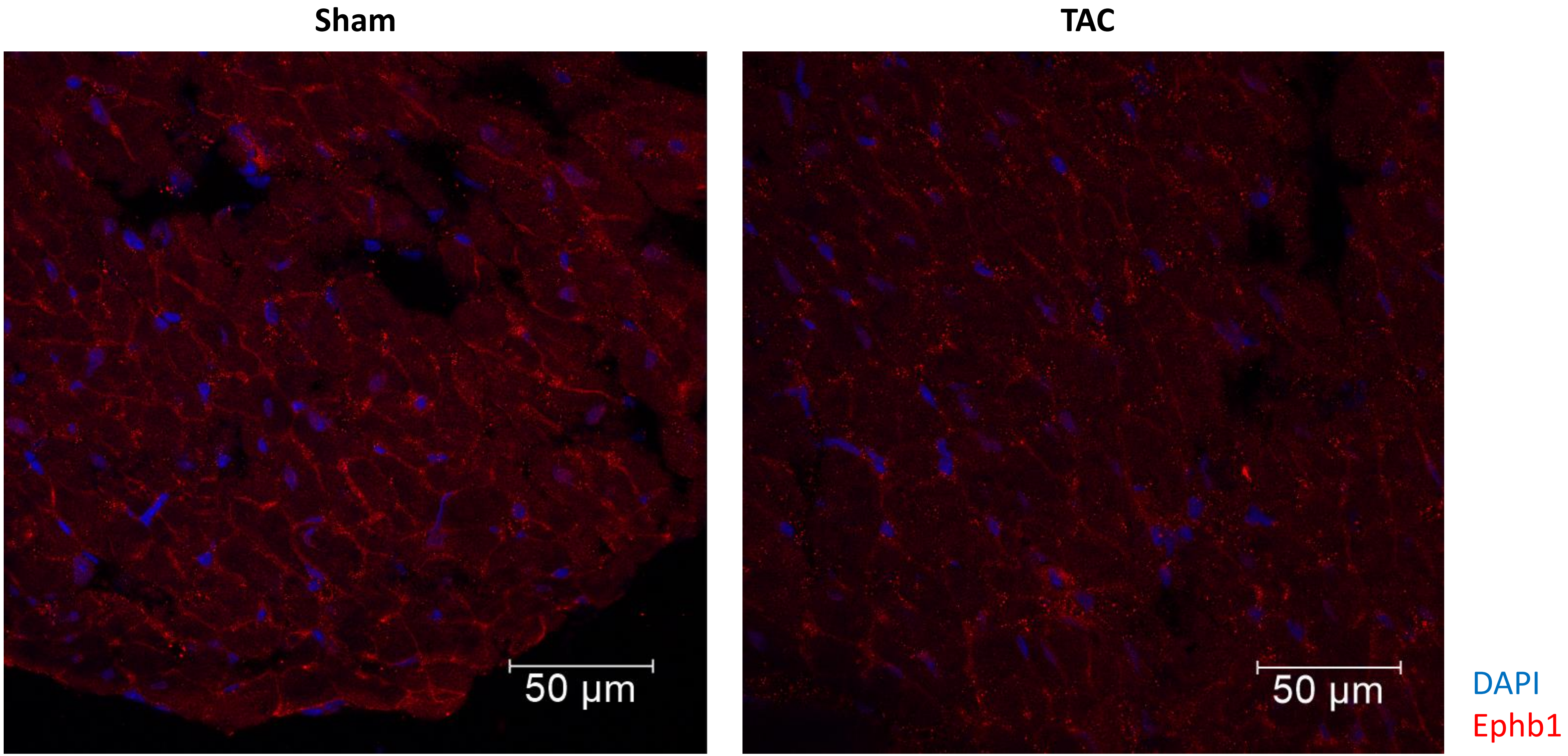

Raw uncropped images from Figure 3j.
