# Supplementary material for: A human cell atlas of the pressure-induced hypertrophic heart
Source: Nat Cardiovasc Res. 2022 Feb 14;1(2):174–85. doi: 10.1038/s44161-022-00019-7 (PMC11357985; doi:10.1038/s44161-022-00019-7)
Supplement: Supplementary file 7 — Raw images for Fig. 4c–e. [file 44161_2022_19_MOESM7_ESM.pdf]

UNCROPPED WESTERNBLOT FILES NAMED:

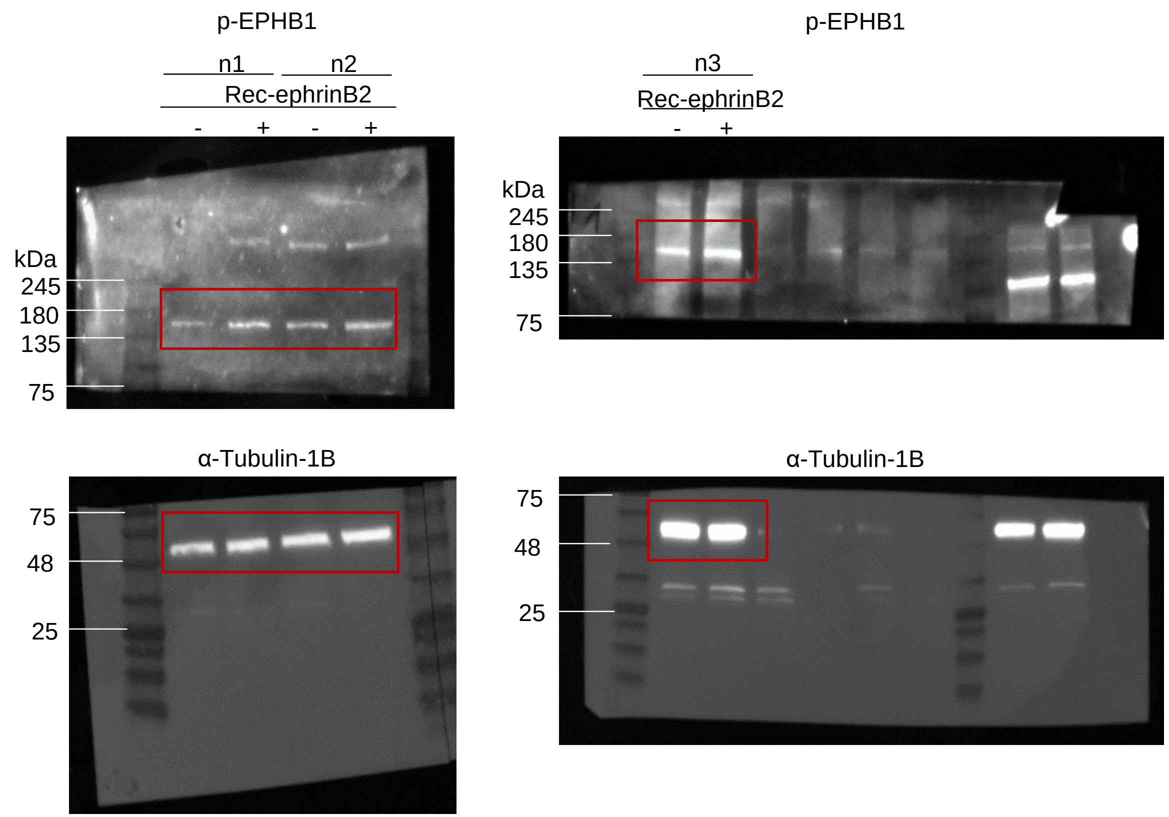

Supplementary Figure 1: **Uncropped Scans of the cropped Western blots** in n=3 related to Figure 4c; Protein level of phosphorylated EPHB1 in human cardiomyocytes treated with recombinant ephrin-B2 (“+”; 10µg/ml, 15min), compared to non-treated CMs (“-). α-Tubulin-1B served as loading control. The red box indicated the relevant protein band, determined by the company-described size in kDa.

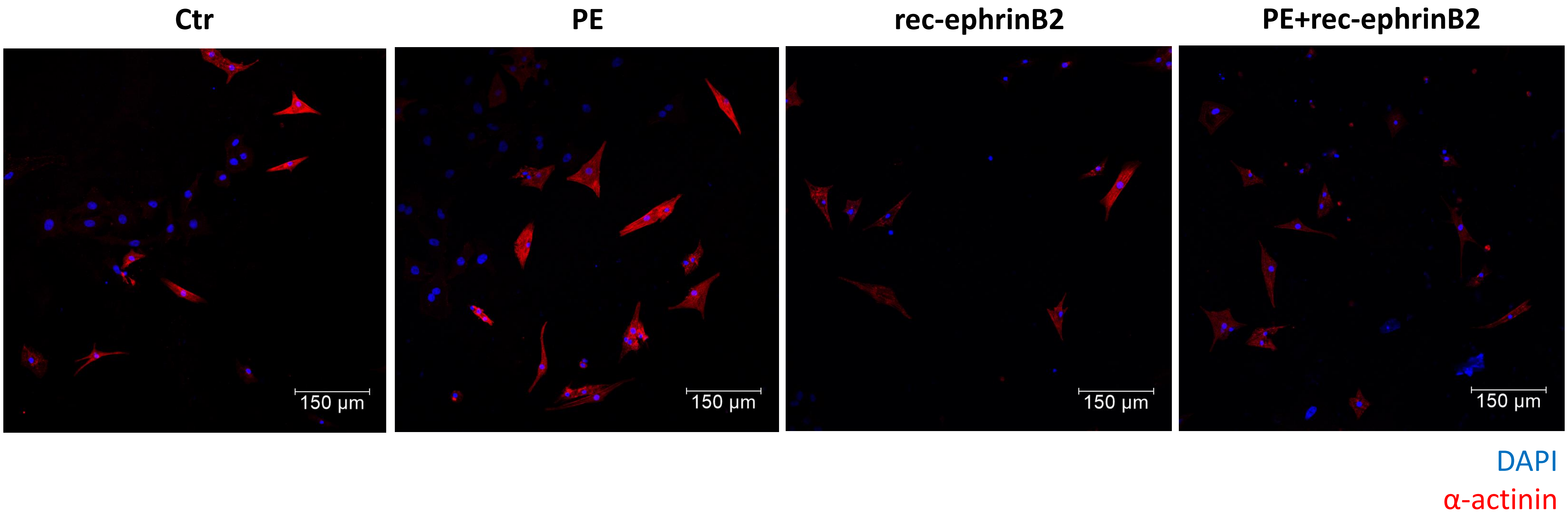

Raw uncropped images from Figure 4d.

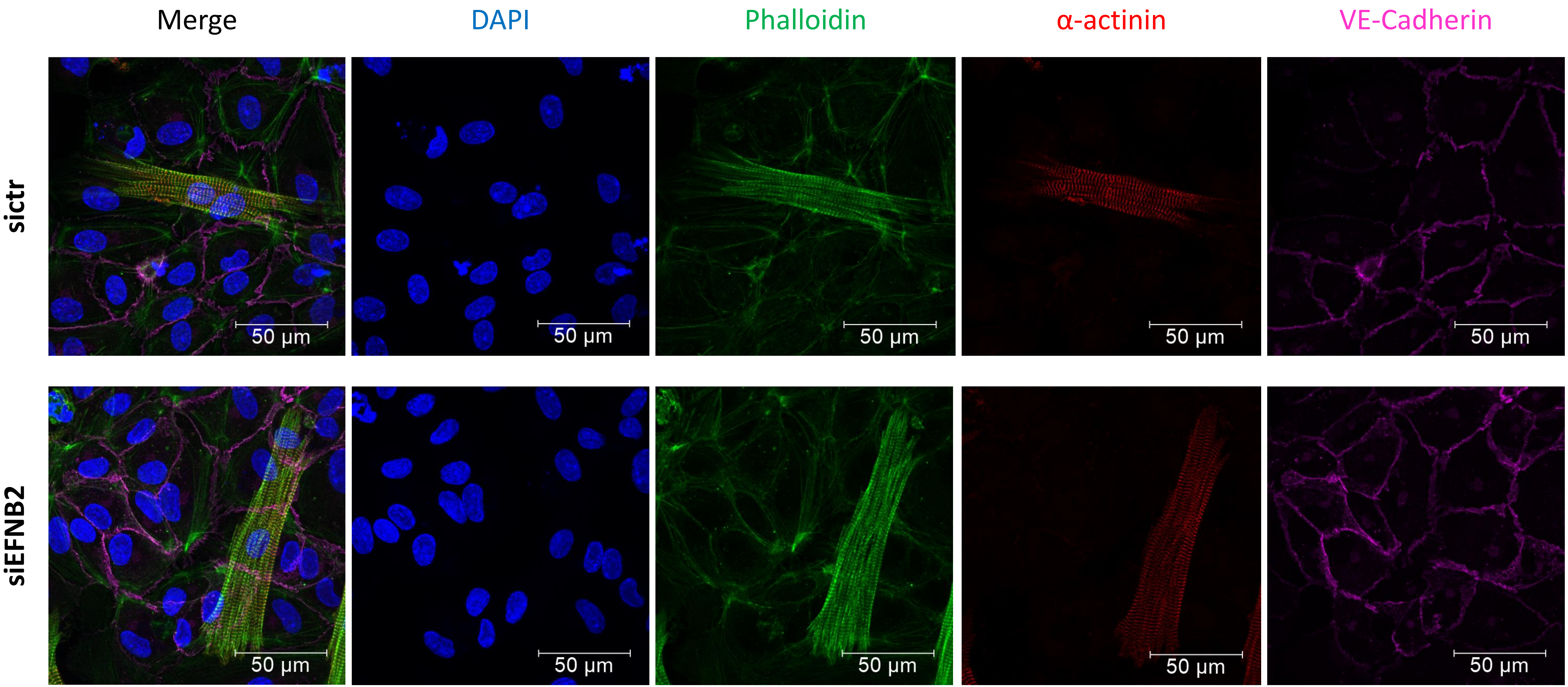

Raw uncropped images from Figure 4e.
