# Supplementary figures and images for: A human cell atlas of the pressure-induced hypertrophic heart
Source: Nat Cardiovasc Res. 2022 Feb 14;1(2):174–85. doi: 10.1038/s44161-022-00019-7 (PMC11357985; doi:10.1038/s44161-022-00019-7)

**Non-Hypertrophied**

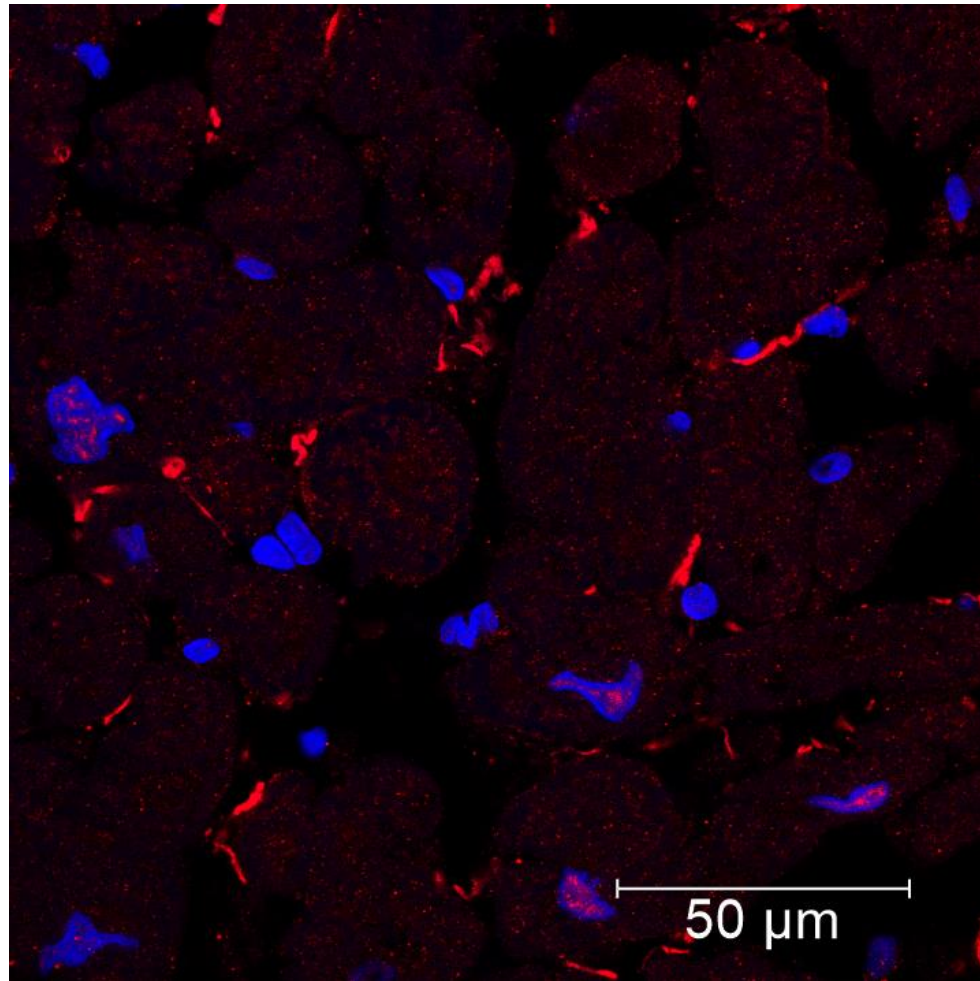

**Hypertrophied**

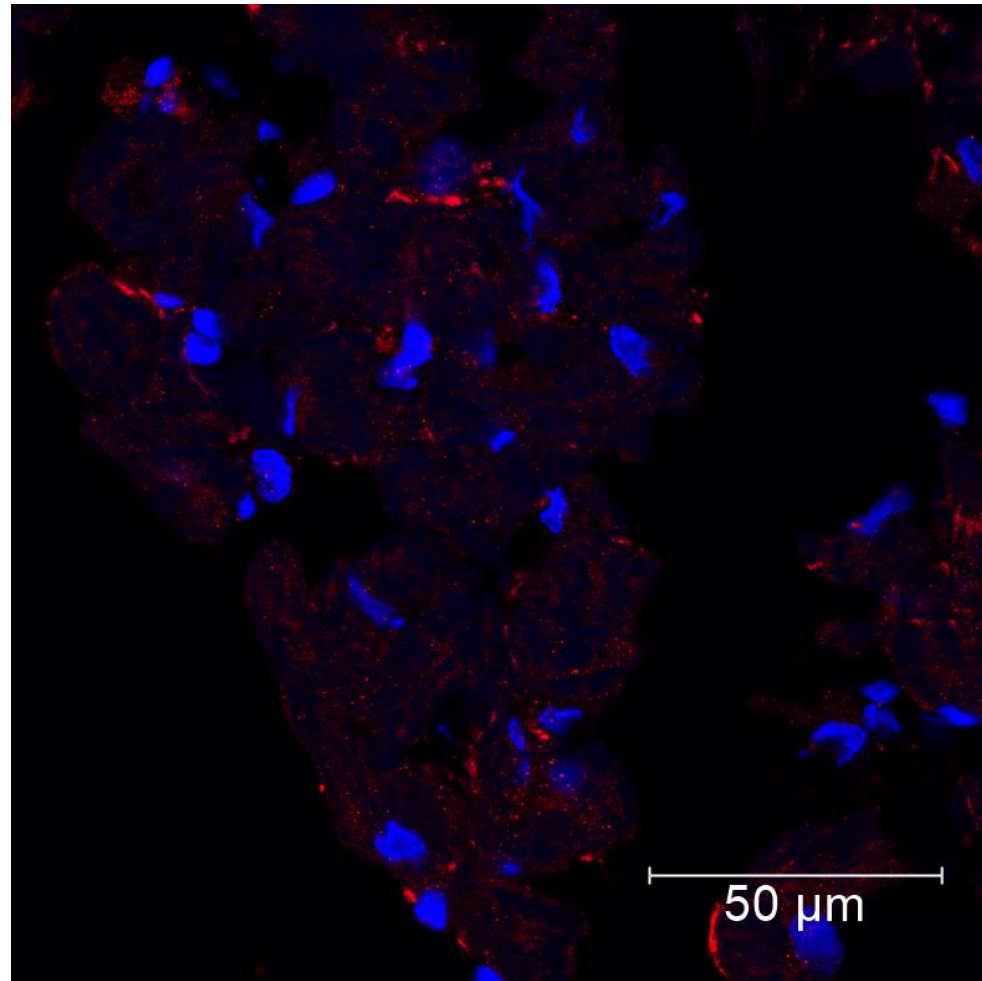

DAPI  
VEGFB

Raw uncropped images from Extended Data Figure 6d.

Supplement: Supplementary file 10 — Raw images for Extended Data Fig. 6d. [file 44161_2022_19_MOESM10_ESM.pdf]

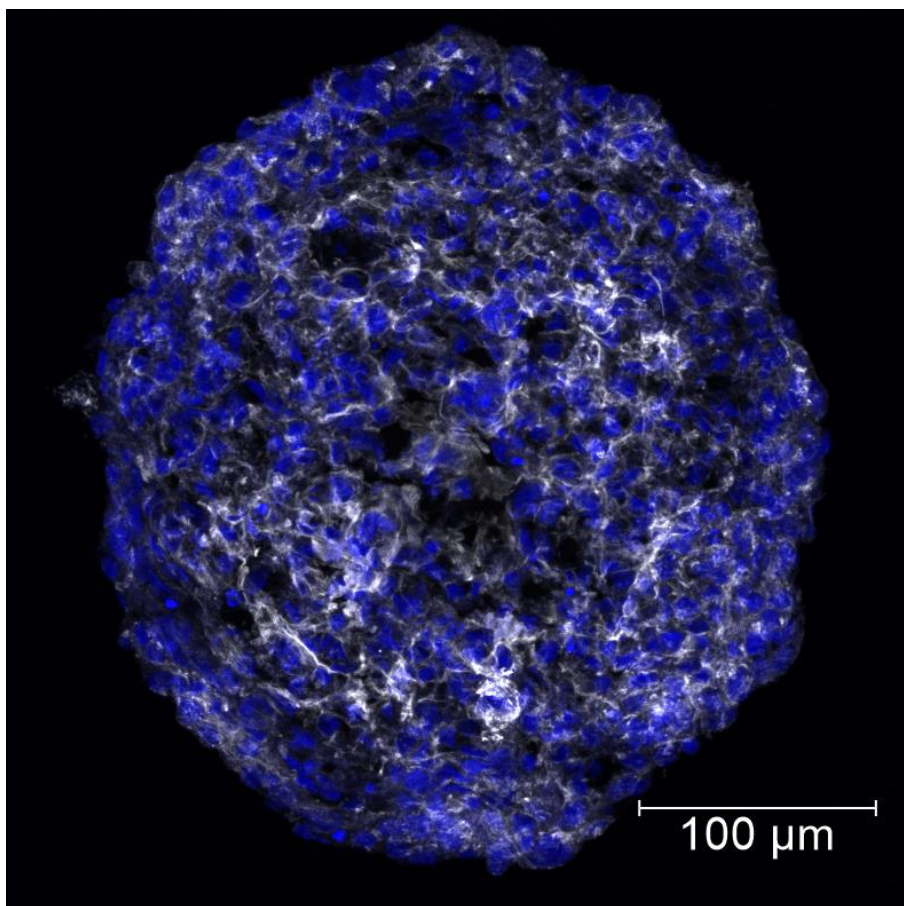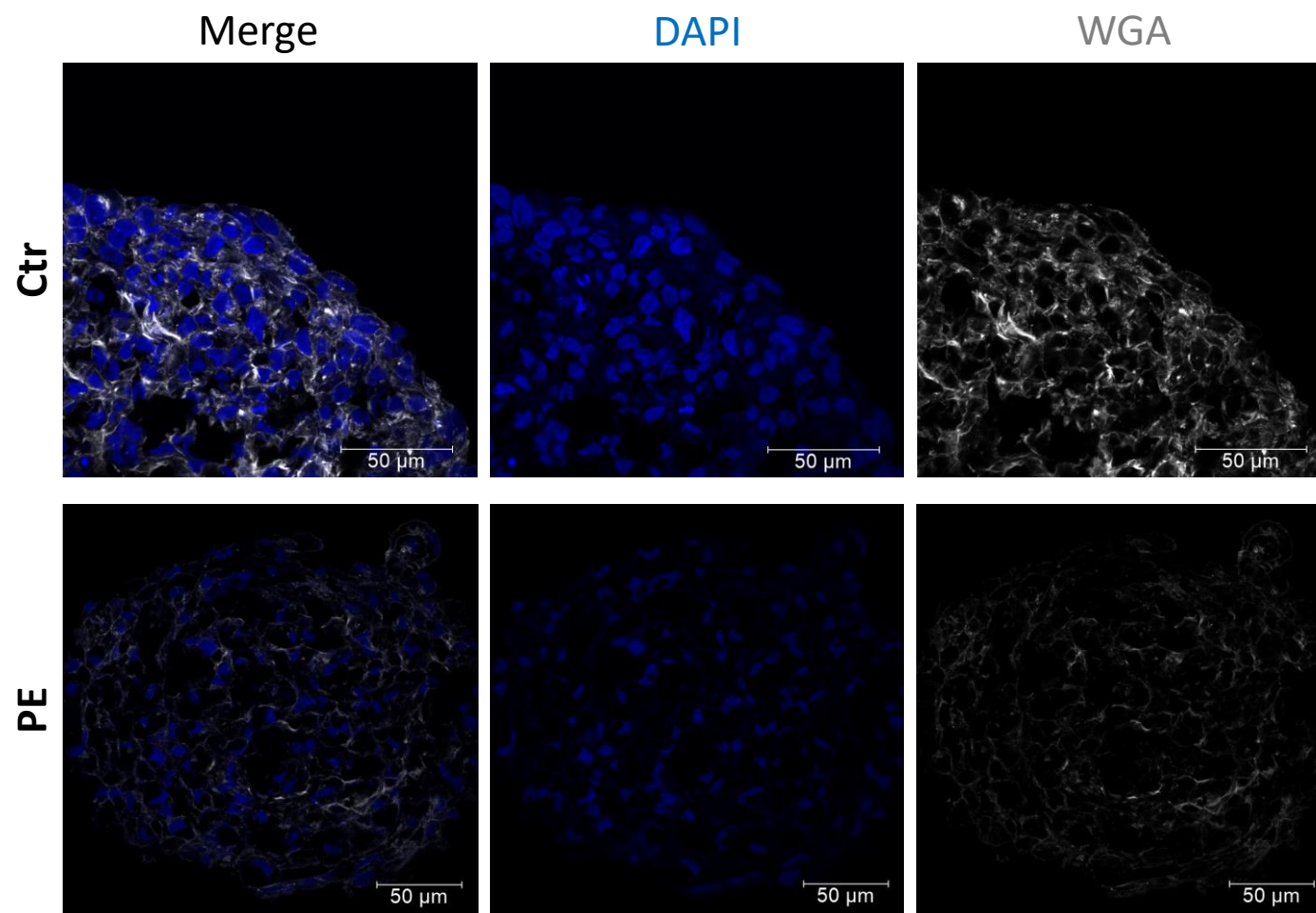

Raw uncropped images from Extended Data Figure 9a.

Supplement: Supplementary file 14 — Raw images for Extended Data Fig. 9a. [file 44161_2022_19_MOESM14_ESM.pdf]
