# Supplementary material for: A human cell atlas of the pressure-induced hypertrophic heart
Source: Nat Cardiovasc Res. 2022 Feb 14;1(2):174–85. doi: 10.1038/s44161-022-00019-7 (PMC11357985; doi:10.1038/s44161-022-00019-7)
Supplement: Supplementary file 12 — Raw image for Extended Data Fig. 8f. [file 44161_2022_19_MOESM12_ESM.pdf]

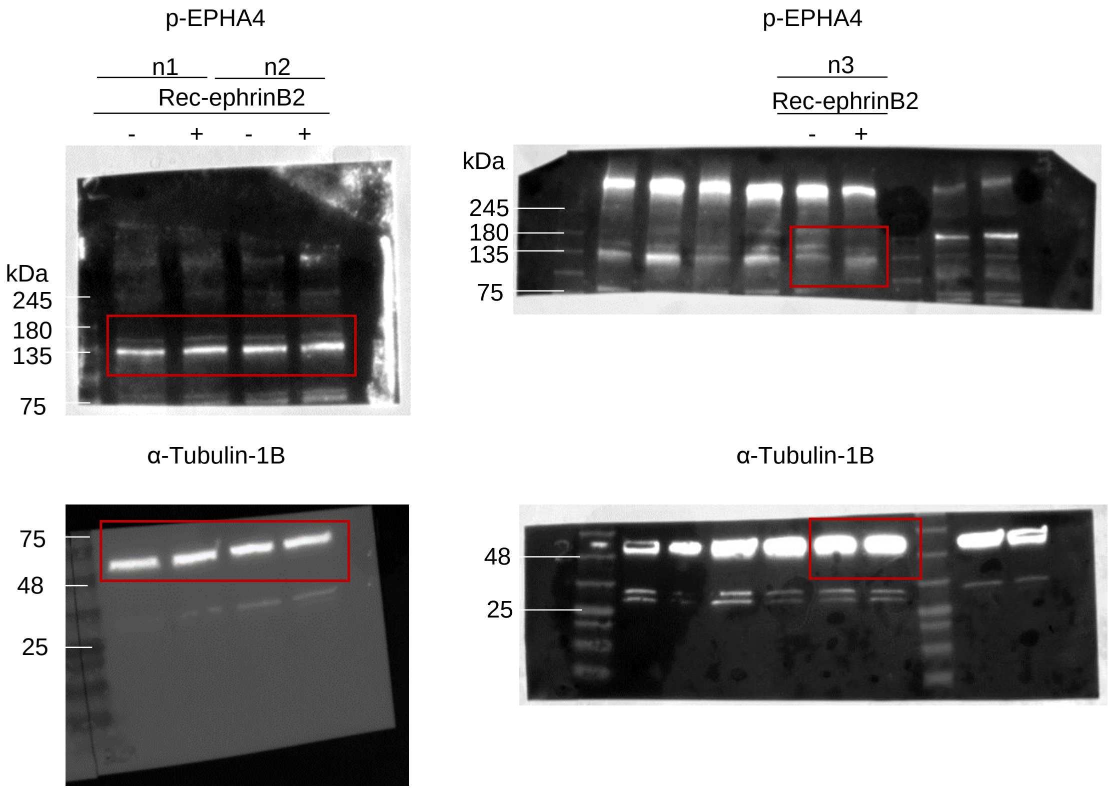

Source Data ED Fig. 8f: **Uncropped Scans of the cropped Western blots** in n=3 related to Extended Data Figure 8f. Protein level of phosphorylated EPHA4 in human cardiomyocytes treated with recombinant ephrin-B2 ("+"; 10µg/ml, 15min), compared to non-treated CMs ("-). α-Tubulin-1B served as loading control. The red box indicated the relevant protein band, determined by the company-described size in kDa.
